# Decoding the molecular mechanisms of pyroptosis and its therapeutic development prospects

**DOI:** 10.3389/fcell.2026.1867415

**Published:** 2026-08-13

**Authors:** Yi Shen, Qiangqiang Zheng, Yang Yuan, Wei Chen, Min Liang, Feng Jian, Maohua Li, Yunfeng Zhou

**Affiliations:** Department of Thoracic Surgery, West China School of Public Health and West China Fourth Hospital, Sichuan University, Chengdu, Sichuan, China

**Keywords:** gasdermin, inflammasome, nanomaterials, pyroptosis, stem cell exosomes, therapeutic target, tumor immunity

## Abstract

Pyroptosis is an emerging form of inflammatory programmed cell death primarily mediated by the gasdermin protein family, characterized by cell swelling, membrane pore formation, and the release of pro-inflammatory cytokines such as IL-1β and IL-18. This mode of cell death has garnered significant attention due to its critical involvement in a variety of diseases, including cancer, cardiovascular disorders, autoimmune diseases, and metabolic syndromes, positioning it as a promising therapeutic target. Despite advances, the complexity of pyroptosis regulation and its dual roles in disease pathogenesis present challenges that necessitate comprehensive understanding. This review systematically summarizes the molecular regulatory mechanisms of pyroptosis, encompassing classical and non-classical inflammasome pathways, activation and modulation of gasdermin proteins, and the intricate signaling networks involved. We further highlight the dualistic roles of pyroptosis in tumor development, immune modulation, and chronic inflammatory conditions. Incorporating the latest research progress, we explore emerging therapeutic strategies targeting pyroptosis, including small molecule inhibitors, nanomaterials, stem cell-derived exosomes, and gene regulation technologies, critically evaluating their clinical applicability and limitations. By providing an integrative overview of pyroptosis-related mechanisms and innovative treatment approaches, this article aims to offer a theoretical foundation and future research direction for precision medicine and novel drug development.

## 1 Introduction

Pyroptosis, a form of programmed inflammatory cell death, has emerged as a distinct and critical mode of regulated cell death characterized by the formation of plasma membrane pores, cell swelling, lysis, and the release of proinflammatory cytokines such as interleukin-1β (IL-1β) and interleukin-18 (IL-18). Unlike apoptosis, which is generally non-inflammatory and immunologically silent, pyroptosis actively promotes inflammation and immune responses, distinguishing it from other cell death modalities including necrosis and necroptosis (Sollberger, 2022; Burdette et al., 2021). The execution of pyroptosis is primarily mediated by the gasdermin (GSDM) family of pore-forming proteins, with gasdermin D (GSDMD) being the most extensively studied member. Upon cleavage by inflammatory caspases such as caspase-1, -4, -5, and -11, the N-terminal domain of GSDMD translocates to the plasma membrane, oligomerizes, and forms large pores, leading to cell membrane rupture and the release of intracellular inflammatory mediators (Liu and Lieberman, 2024; Li and Guo, 2025). This process is tightly regulated by inflammasomes, multi-protein complexes that sense pathogen-associated molecular patterns (PAMPs) and damage-associated molecular patterns (DAMPs), initiating caspase activation and subsequent pyroptosis (Li et al., 2025a).

Recent advances have expanded the understanding of pyroptosis beyond canonical inflammasome-dependent pathways to include non-canonical and caspase-3/8-mediated mechanisms, as well as inflammasome-independent activation of GSDMs (Burdette et al., 2021; Liu and Lieberman, 2024). The complexity of pyroptotic signaling is further underscored by the involvement of post-translational modifications such as phosphorylation, ubiquitination, and acetylation, which modulate GSDM activity and pyroptosis execution (Oladapo et al., 2024; Chen Y. et al., 2024; Li Y. et al., 2022). Furthermore, non-coding RNAs (ncRNAs), including microRNAs (miRNAs) and long non-coding RNAs (lncRNAs), have been identified as critical regulators of pyroptosis-related gene expression, influencing disease pathogenesis and therapeutic responses (Wu and Ji, 2026; Stoess et al., 2023).

Pyroptosis plays a pivotal role in diverse physiological and pathological contexts, including host defense against microbial infections, inflammatory diseases, and cancer. In infectious diseases, pyroptosis contributes to the elimination of intracellular pathogens by destroying their replicative niches and promoting robust inflammatory responses (Wang Z. et al., 2022; Booty and Bryant, 2022). However, dysregulated pyroptosis can exacerbate tissue damage and chronic inflammation, contributing to the pathogenesis of autoimmune disorders, metabolic diseases, and neurodegenerative conditions (Chai R. et al., 2023; Feng et al., 2022; Huang et al., 2022a). For example, in diabetic nephropathy (DN) and diabetic cardiomyopathy, pyroptosis induced by hyperglycemia and inflammasome activation leads to renal and cardiac injury, respectively (Al Mamun et al., 2021a; Cai Z. et al., 2022). Similarly, in neurological diseases such as Alzheimer’s disease and stroke, pyroptosis-mediated neuroinflammation exacerbates neuronal loss and disease progression (Huang et al., 2022a; Yang et al., 2021).

In the realm of oncology, pyroptosis exhibits a dualistic role. On one hand, pyroptotic cell death can suppress tumor growth by inducing immunogenic cell death, enhancing dendritic cell (DC) maturation, and promoting cytotoxic T lymphocyte infiltration, thereby potentiating antitumor immunity (Yang F. et al., 2022; Johnson and Cui, 2025; Hou et al., 2023). On the other hand, chronic pyroptosis-driven inflammation can foster a tumor-promoting microenvironment that facilitates cancer progression and metastasis (Wang L. et al., 2021; Yin H. et al., 2024). The GSDM family proteins, particularly GSDMD and gasdermin E (GSDME), have been implicated in mediating pyroptosis in various cancers, including lung, breast, ovarian, melanoma, and gastrointestinal tumors (Hou et al., 2023; Huang et al., 2022b; Wu J. et al., 2022; Wu X. et al., 2022). Therapeutic strategies aimed at inducing pyroptosis in tumor cells have shown promise in overcoming resistance to conventional therapies and enhancing the efficacy of immunotherapies such as immune checkpoint inhibitors (ICIs) (Hsu et al., 2021; Bao et al., 2024; Xie et al., 2025).

Technological advances in nanomedicine have opened new avenues for the precise modulation of pyroptosis to achieve therapeutic benefits. Nanoparticle-based delivery systems have been engineered to induce pyroptosis selectively within tumors, thereby amplifying antitumor immune responses while minimizing systemic toxicity (Ji et al., 2024; Qi et al., 2025; Trois et al., 2025). These nanoplatforms can co-deliver pyroptosis inducers and immunomodulators, facilitating combinatorial therapies that remodel the tumor immune microenvironment (TIME) and improve clinical outcomes (Huang et al., 2023; Yu et al., 2025). Moreover, nanomaterials have been employed to inhibit pyroptosis in inflammatory diseases, offering novel therapeutic strategies for conditions such as rheumatoid arthritis (RA), atherosclerosis, and acute pancreatitis (Wu and Ji, 2026; Chai Q. et al., 2023; Wu S. et al., 2025).

The expanding knowledge of pyroptosis molecular mechanisms has also identified novel therapeutic targets, including inflammasome components, caspases, and GSDMs themselves. Small molecule inhibitors, natural compounds, and biologics targeting these molecules have demonstrated efficacy in preclinical models of inflammatory and metabolic diseases, as well as cancer (Xu L. et al., 2025; Cai et al., 2024; Zhang X. et al., 2025). For instance, inhibitors of NLRP3 inflammasome activation and GSDMD pore formation have shown promise in mitigating DN, myocardial ischemia-reperfusion injury, and other pathological conditions (Al Mamun et al., 2021b; Liu Y. et al., 2024; Phungphong et al., 2025). Furthermore, modulation of pyroptosis by post-translational modifications and ncRNAs offers additional layers of therapeutic intervention (Chen Y. et al., 2024; Wu and Ji, 2026).

In summary, pyroptosis represents a unique and versatile form of programmed cell death with significant implications in health and disease. Its intricate molecular regulation, involvement in diverse pathologies, and the potential for therapeutic modulation underscore its importance as a frontier in biomedical research. This review aims to provide a comprehensive and systematic overview of the molecular mechanisms underlying pyroptosis, its complex roles in various diseases including cancer, cardiovascular, metabolic, neurological, and inflammatory disorders, and the current advances in leveraging pyroptosis pathways for therapeutic development. By integrating recent discoveries and emerging technologies such as nanomedicine and RNA-based therapeutics, this work seeks to inform future research directions and clinical translation efforts targeting pyroptosis.

## 2 Pyroptosis: molecular mechanisms, disease relevance, and therapeutic applications

### 2.1 Overview of the molecular mechanisms of pyroptosis

#### 2.1.1 Definition and biological characteristics of pyroptosis

Pyroptosis is a distinct form of inflammatory programmed cell death characterized by cell swelling, plasma membrane rupture, and the release of pro-inflammatory mediators. Unlike apoptosis, which is generally immunologically silent, pyroptosis actively initiates inflammatory responses and serves as an important component of innate immune defense (Sollberger, 2022; Yang F. et al., 2022; Al Mamun et al., 2021b). While this process contributes to pathogen clearance and tissue protection, excessive or dysregulated pyroptosis may promote chronic inflammation and tissue injury (Liu and Lieberman, 2024). Morphologically, pyroptosis is distinguished by rapid cellular swelling and membrane rupture, whereas apoptosis is characterized by cellular shrinkage and preservation of membrane integrity (Zhang L. et al., 2021; Yu et al., 2021).

Pyroptosis differs mechanistically from necrosis, which is traditionally viewed as an unregulated form of cell death resulting from acute injury, lacking the specific molecular pathways that characterize pyroptosis. While necrosis also leads to cell swelling and membrane rupture, pyroptosis is a regulated process involving specific signaling cascades and proteolytic events (Bertheloot et al., 2021). Importantly, pyroptosis can be triggered via canonical and non-canonical pathways: the canonical pathway involves caspase-1 activation through inflammasomes, whereas the non-canonical pathway involves caspase-4/5/11 responding to intracellular lipopolysaccharide (LPS) (Burdette et al., 2021). Additionally, caspase-3 and caspase-8 have been implicated in pyroptosis through cleavage of GSDME and GSDMD, respectively, highlighting the complexity and crosstalk between pyroptosis and other cell death modalities such as apoptosis (Wang et al., 2020; Shi et al., 2023).

Biologically, pyroptosis serves as a critical innate immune mechanism, eliminating infected or damaged cells and alerting the immune system through the release of inflammatory mediators. This process is essential in controlling infections and shaping the tumor microenvironment (TME), where pyroptosis can have dual roles, either suppressing tumor growth by killing cancer cells or promoting tumor progression through chronic inflammation (Al Mamun et al., 2021a; Ning et al., 2022; Zhou et al., 2022a; Wei et al., 2025). The inflammatory nature of pyroptosis distinguishes it from apoptosis and necrosis and underscores its significance in various diseases, including infections, autoimmune disorders, cardiovascular diseases, and cancers (Johnson and Cui, 2025; Wang et al., 2020; Sordi et al., 2021).

#### 2.1.2 Core roles of the GSDM family in pyroptosis

GSDM family proteins, notably GSDMD and GSDME, serve as the pivotal executioners of pyroptosis, a highly inflammatory form of programmed cell death. Structurally, GSDMs share a conserved architecture composed of an N-terminal domain responsible for pore formation and a C-terminal domain that autoinhibits the pore-forming activity in the resting state. Activation of GSDMs is tightly regulated by proteolytic cleavage, which separates the N-terminal domain from the inhibitory C-terminal domain, unleashing the pore-forming capacity essential for pyroptotic cell death. This cleavage liberates the GSDMD N-terminal fragment (GSDMD-N), which oligomerizes and inserts into the plasma membrane to form transmembrane pores. Proteolytic cleavage releases the N-terminal fragment, enabling membrane pore formation and subsequent pyroptotic cell death (Jiang et al., 2020).

The molecular mechanism underlying GSDM pore formation involves the N-terminal domain binding to specific phospholipids on the inner leaflet of the plasma membrane, followed by oligomerization into large, ring-shaped pores approximately 10–20 nm in diameter. These pores compromise membrane integrity, leading to cell swelling, membrane rupture, and the release of intracellular inflammatory mediators. The pore formation is a multistep process beginning with membrane targeting, followed by insertion and oligomerization, which collectively disrupt the lipid bilayer architecture (Shao, 2021; Huston et al., 2023). Notably, the pore-forming activity of GSDMs is not only a terminal event leading to cell death but can also be reversible under certain conditions, allowing cells to modulate inflammatory responses without immediate lysis (Zhao et al., 2025). Additionally, post-translational modifications, such as succination of GSDMD by fumarate, can inhibit its cleavage and pore formation, highlighting an additional layer of regulation that controls pyroptotic activity and inflammatory outcomes (Humphries et al., 2020).

Collectively, the GSDM family constitutes a versatile and evolutionarily conserved system for executing pyroptosis through structurally and mechanistically defined pore formation, which is central to inflammatory responses and holds significant therapeutic potential in inflammatory diseases and cancer (Bai et al., 2025; Ouyang et al., 2023).

#### 2.1.3 Activation of inflammasomes and pyroptosis signaling

Inflammasomes are cytosolic multiprotein complexes that serve as critical innate immune sensors detecting PAMPs and DAMPs. Among the various inflammasomes, NLRP3 and AIM2 (absent in melanoma 2) are well-characterized for their roles in initiating pyroptosis, a form of inflammatory programmed cell death. The assembly of inflammasomes typically begins with the recognition of activating stimuli by sensor proteins such as NLRP3 or AIM2. Upon activation, these sensors oligomerize and recruit the adaptor protein ASC (apoptosis-associated speck-like protein containing a CARD), which facilitates the recruitment and activation of pro-caspase-1. This leads to the formation of a multiprotein inflammasome complex that acts as a platform for caspase-1 activation. Activated caspase-1 then cleaves pro-inflammatory cytokine precursors pro-IL-1β and pro-IL-18 into their mature forms and also cleaves GSDMD, releasing its GSDMD-N that forms pores in the plasma membrane, thereby executing pyroptosis and enabling cytokine release (Li and Jiang, 2023; Liang et al., 2020). The NLRP3 inflammasome is unique in its ability to respond to a broad spectrum of stimuli, including microbial infections, metabolic stress, and crystalline substances. Its activation involves complex regulatory mechanisms such as mitochondrial reactive oxygen species (mtROS) generation, potassium efflux, and lysosomal damage, which converge to induce conformational changes that promote inflammasome assembly (Duan et al., 2020; Qiu et al., 2022). AIM2, on the other hand, senses cytosolic double-stranded DNA and assembles inflammasomes that similarly activate caspase-1. Recent studies employing optogenetic tools have demonstrated that inflammasome activation leads to distinct phases of cell swelling and membrane rupture during pyroptosis, underscoring the dynamic nature of inflammasome-mediated cell death (Nadjar et al., 2024). Furthermore, novel regulatory proteins such as RACK1 and CANE have been identified to modulate NLRP3 inflammasome assembly and activity, highlighting the complexity of inflammasome regulation (Duan et al., 2020; Kaneko et al., 2024). Overall, the assembly and activation of inflammasomes like NLRP3 and AIM2 constitute a tightly regulated process that integrates diverse cellular signals to initiate pyroptosis and pro-inflammatory cytokine release, essential for host defense and homeostasis.

The canonical and non-canonical inflammasome pathways represent two distinct but interconnected signaling cascades that culminate in pyroptosis and inflammatory cytokine release. The canonical pathway is initiated by inflammasome sensors such as NLRP3, AIM2, or NLRC4, which recruit and activate caspase-1 through the adaptor ASC. Activated caspase-1 cleaves pro-IL-1β and pro-IL-18 to their mature forms and processes GSDMD, whose GSDMD-N forms membrane pores leading to pyroptotic cell death (Li and Jiang, 2023; Liang et al., 2020). In contrast, the non-canonical pathway involves direct sensing of cytosolic LPS by inflammatory caspases caspase-4 and caspase-5 in humans, and caspase-11 in mice. These caspases do not require upstream inflammasome sensors for activation; instead, they directly bind LPS, undergo autoproteolysis, and cleave GSDMD to induce pyroptosis (Wright et al., 2022; Wu J. et al., 2023). Notably, non-canonical inflammasome activation can also promote secondary activation of the canonical NLRP3 inflammasome, linking the two pathways functionally (Wu J. et al., 2023). While both pathways converge on GSDMD cleavage and pore formation, they differ in their upstream sensing mechanisms and caspase utilization. The canonical pathway is more versatile, responding to a wide range of stimuli via various sensors, whereas the non-canonical pathway specifically detects intracellular LPS, playing a critical role in defense against gram-negative bacterial infections and in sepsis pathogenesis (Wright et al., 2022; Oh et al., 2025). Furthermore, zinc homeostasis has been shown to regulate both canonical and non-canonical inflammasome activation by inhibiting caspase-1 and caspase-4/5/11 activities, indicating additional layers of regulation (Gong X. et al., 2024). Importantly, cell type-specific differences exist; for example, neutrophils can resist pyroptotic lysis despite inflammasome activation, allowing them to release IL-1β while maintaining viability, highlighting nuanced regulation of pyroptosis (Dubyak et al., 2023). Therapeutically, targeting either pathway holds promise for treating inflammatory diseases, sepsis, and cancer, with ongoing research exploring inhibitors that can broadly suppress inflammasome activation (Anderson et al., 2024; Seo et al., 2025). In summary, the canonical and non-canonical inflammasome pathways represent complementary arms of innate immunity, distinguished by their caspase usage and activation mechanisms, yet interconnected through shared downstream effectors and biological outcomes.

#### 2.1.4 Other regulatory mechanisms: caspase-3/8 and granzyme-mediated pathways

Apoptotic caspases, particularly caspase-3 and caspase-8, have emerged as critical regulators intersecting with pyroptotic pathways, revealing a complex crosstalk between apoptosis and pyroptosis. Traditionally, pyroptosis was primarily associated with inflammatory caspases such as caspase-1, -4, -5, and -11, which cleave GSDMD to form membrane pores. However, recent studies have demonstrated that caspase-3 and caspase-8, key executors of apoptosis, can also induce pyroptosis through cleavage of distinct GSDM family members, especially GSDME and GSDMD, respectively. For instance, caspase-3 cleaves GSDME, liberating its N-terminal domain that forms membrane pores, thereby converting apoptotic signals into pyroptotic cell death characterized by cell swelling and inflammatory cytokine release (Liu M. et al., 2025; Zheng et al., 2021). This mechanism has been implicated in diverse pathological contexts, including neurodegenerative diseases such as Alzheimer’s disease, where elevated caspase-8 expression activates caspase-3 and triggers GSDME-dependent pyroptosis, exacerbating neuroinflammation and cell death (Liu M. et al., 2025). Similarly, caspase-8 can directly cleave GSDMD, inducing pyroptosis independently of caspase-1, as observed in macrophages infected with pathogens like *Yersinia* or stimulated with LPS and TAK1 inhibitors (Zheng et al., 2021; Ma et al., 2025). Moreover, caspase-8 activation is not limited to pathogen responses but also plays a role in sterile inflammation and cancer. For example, α-ketoglutarate induces pyroptosis via caspase-8-mediated cleavage of GSDMC in tumor cells, linking metabolic cues to inflammatory cell death and tumor suppression (Zhang J. Y. et al., 2021). The interplay between caspase-8 and caspase-3 is further highlighted during influenza A virus infection, where caspase-8 autoprocessing activates caspase-3 to suppress GSDMD-mediated pyroptosis, balancing cell death modalities to modulate inflammation and host defense (Wang Y. et al., 2021). This crosstalk underscores the dual role of caspase-3 and caspase-8 as molecular switches that can direct cells towards apoptosis or pyroptosis depending on cellular context and stimuli, thereby fine-tuning inflammatory responses. Additionally, caspase-8’s involvement in inflammasome-associated cell death pathways suggests it can mediate apoptosis, pyroptosis, or necroptosis, contributing to a broader regulated cell death network termed PANoptosis (Song K. et al., 2025; Tsuchiya, 2020). Therapeutically, targeting caspase-3/8-mediated pyroptosis pathways offers promising avenues for diseases characterized by excessive inflammation or defective cell death, including cancer, neurodegeneration, and infectious diseases. For example, inhibition of caspase-8 attenuates pyroptosis and inflammation in bronchopulmonary dysplasia models, improving alveolar development and vascular remodeling (Huang et al., 2025). In summary, apoptotic caspases caspase-3 and caspase-8 serve as pivotal regulators bridging apoptosis and pyroptosis, orchestrating cell fate decisions through GSDM cleavage and providing novel targets for modulating inflammatory cell death in various diseases.

Granzyme B (GzmB), a serine protease secreted by cytotoxic lymphocytes such as natural killer (NK) cells and cytotoxic T lymphocytes (CTLs), has been identified as a potent inducer of pyroptosis through direct cleavage of GSDM family proteins, thereby linking immune effector functions to inflammatory cell death. Traditionally recognized for its role in inducing apoptosis via caspase activation within target cells, GzmB has recently been shown to cleave GSDME, triggering pyroptosis characterized by membrane pore formation, cell swelling, and release of proinflammatory cytokines (Tan et al., 2025; Wang M. et al., 2026). This GzmB -mediated pyroptotic pathway enhances immune-mediated tumor clearance by converting target cancer cells into proinflammatory entities that recruit and activate additional immune cells. For instance, a novel immunotoxin combining an HPV16 E7-specific affibody with GzmB (ZHPV16E7-GrB) induces pyroptosis in HPV16-positive cervical cancer cells by directly cleaving GSDME, independent of caspase-3 activation, resulting in membrane rupture and release of inflammatory cytokines that potentiate antitumor immunity (Tan et al., 2025). Furthermore, GzmB expression is upregulated in pathological conditions such as renal fibrosis, where its knockdown reduces pyroptosis and fibrosis by inhibiting NLRP3 inflammasome activation and downstream JAK2/STAT3 signaling, highlighting its role beyond cytotoxicity in modulating inflammatory responses (Wang M. et al., 2026). The GzmB-GSDME axis also contributes to the immune microenvironment remodeling in cancers such as triple-negative breast cancer (TNBC), where pyroptosis induction enhances CD8^+^ T cell infiltration and GzmB presence, amplifying antitumor immunity (Yang et al., 2024). The GzmB-mediated pyroptosis pathway represents a critical intersection between immune cytotoxicity and inflammatory cell death, offering therapeutic potential for enhancing antitumor immunity and controlling inflammatory diseases. Targeting this pathway could improve immunotherapy efficacy by promoting immunogenic cell death and overcoming tumor immune evasion. In conclusion, GzmB acts as a key mediator of pyroptosis in immune surveillance and pathology, expanding the functional repertoire of cytotoxic lymphocytes in host defense and disease modulation.

#### 2.1.5 NcRNAs and epigenetic regulation

LncRNAs and circular RNAs (circRNAs) have emerged as critical regulators of pyroptosis by modulating the expression of genes involved in this inflammatory form of programmed cell death. LncRNAs, typically over 200 nucleotides in length and lacking protein-coding capacity, exert diverse molecular functions including acting as molecular sponges for miRNAs, scaffolding epigenetic modifiers, and guiding chromatin remodeling complexes to specific genomic loci. For instance, in cancer contexts such as HCC and colorectal cancer (CRC), lncRNAs have been shown to influence pyroptosis by regulating the expression of inflammasome components and GSDMs, thereby affecting tumor cell proliferation, invasion, and treatment resistance (Luo et al., 2026; Taha et al., 2025). Similarly, circRNAs, characterized by covalently closed loop structures, regulate pyroptosis-related genes primarily through competing endogenous RNA (ceRNA) networks, sequestering miRNAs that target pyroptosis mediators, thus fine-tuning the pyroptotic response. In cardiovascular diseases and pulmonary arterial hypertension, ncRNAs including lncRNAs and circRNAs modulate pyroptosis pathways by influencing inflammatory signaling and cell death in vascular cells, which contributes to disease progression (Jiang et al., 2023; Chen C. et al., 2024). Moreover, in immune-mediated diseases such as vasculitis, lncRNAs and circRNAs packaged in extracellular vesicles can regulate pyroptosis and inflammation in target cells, highlighting their role in intercellular communication and immune regulation (Tang et al., 2024). The regulatory capacity of these ncRNAs extends to controlling transcription factors and signaling pathways that govern inflammasome activation, caspase cleavage, and GSDM pore formation, all central to pyroptosis execution. Given their tissue-specific expression and multifaceted regulatory roles, lncRNAs and circRNAs represent promising therapeutic targets and biomarkers for diseases where pyroptosis plays a pathogenic or protective role. Advances in delivery technologies, such as lentiviral vectors and lipid nanoparticles, are facilitating the development of lncRNA-based therapies aimed at modulating pyroptosis for improved clinical outcomes (Fu et al., 2026). Collectively, the intricate regulation of pyroptosis by lncRNAs and circRNAs underscores the complexity of epigenetic control in inflammatory cell death and offers novel avenues for targeted intervention.

**FIGURE 1:**
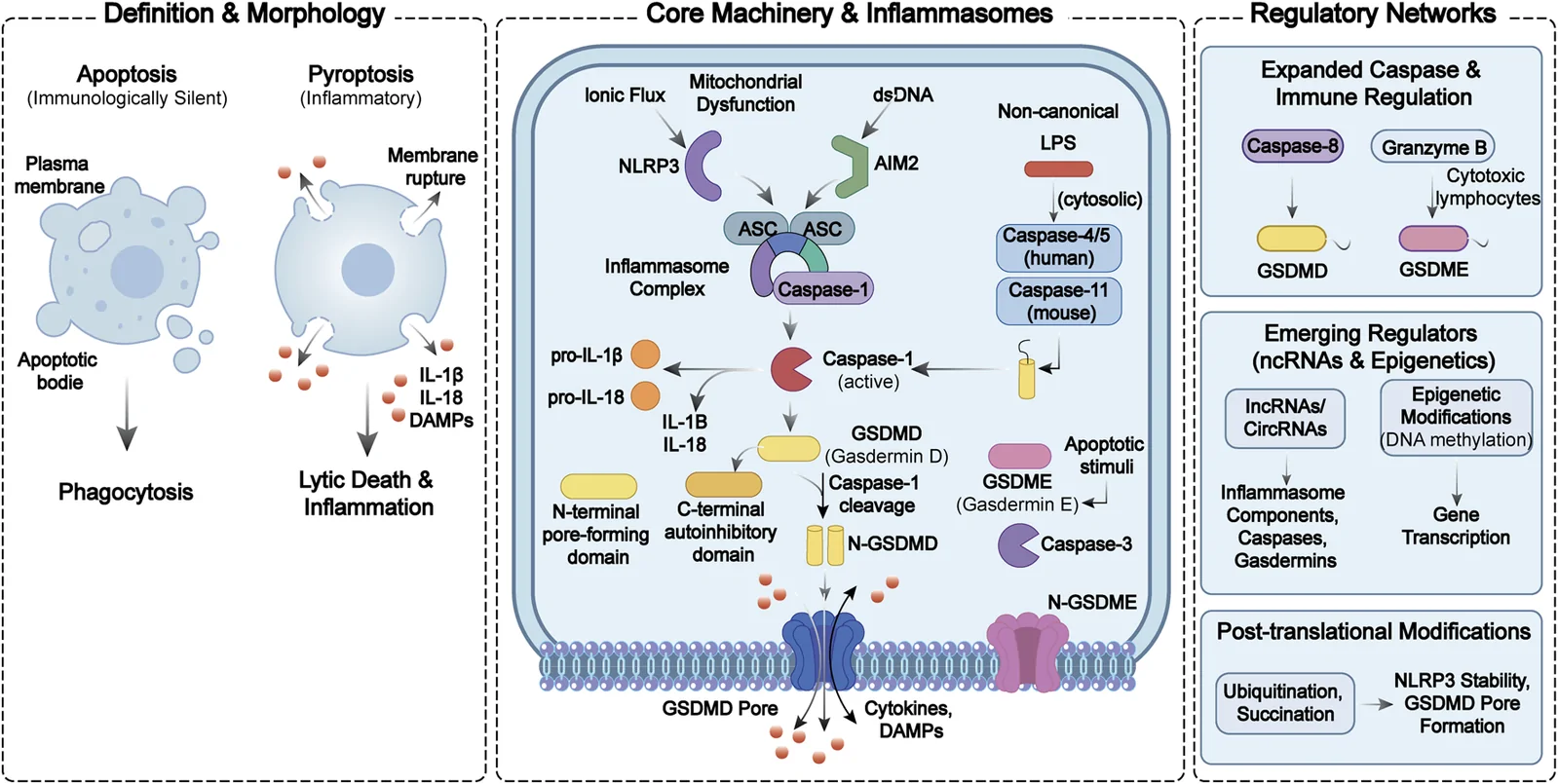
Overview of pyroptosis signaling pathways.

Epigenetic modifications such as DNA methylation and post-translational modifications including ubiquitination critically influence the expression and functional stability of key proteins involved in pyroptosis, thereby modulating the pyroptotic pathway and its biological consequences. DNA methylation, typically involving the addition of methyl groups to cytosine residues in CpG islands, can repress or activate gene transcription depending on the context. Aberrant DNA methylation patterns have been implicated in the dysregulation of inflammasome components such as NLRP3 and caspase-1, which are pivotal for pyroptosis initiation. For example, hypermethylation of promoter regions can silence genes encoding inflammasome sensors or effectors, attenuating pyroptotic responses and influencing disease progression in cancer and inflammatory disorders (Zhang R. N. et al., 2023; Raneros et al., 2021). Conversely, hypomethylation may lead to overexpression of pyroptosis-related genes, contributing to excessive inflammation and tissue damage as observed in sepsis and cardiovascular diseases (Chen C. et al., 2024; Wen et al., 2022). Ubiquitination, a reversible post-translational modification involving the attachment of ubiquitin molecules to lysine residues on target proteins, regulates the stability, localization, and activity of pyroptosis mediators. Key proteins such as NLRP3 undergo ubiquitination that can either mark them for proteasomal degradation or modulate their inflammasome assembly capacity. Deubiquitinases and E3 ubiquitin ligases dynamically control this balance, thereby fine-tuning inflammasome activation and downstream pyroptotic events (He et al., 2025). The crosstalk between DNA methylation and ubiquitination pathways further adds complexity to pyroptosis regulation; epigenetic states can influence the expression of ubiquitin-modifying enzymes, while ubiquitination can affect chromatin modifiers that control DNA methylation landscapes. This multilayered regulation is evident in aging-related diseases where epigenetic reprogramming recalibrates pyroptosis thresholds, impacting tissue homeostasis and disease susceptibility (Liu L. et al., 2025). Understanding these epigenetic and post-translational mechanisms opens new therapeutic possibilities, such as targeting DNA methyltransferases or ubiquitin-proteasome system components to restore balanced pyroptosis in pathological conditions, including cancer, sepsis, and chronic inflammatory diseases (Zhang R. N. et al., 2023; Zhou et al., 2024). Thus, DNA methylation and ubiquitination constitute fundamental regulatory axes that modulate the expression and function of pyroptosis key proteins, shaping the cellular inflammatory response and influencing disease outcomes (Figure 1). The major activation pathways and regulatory layers governing pyroptosis are summarized in Table 1, highlighting the convergence of diverse upstream signals on GSDM-mediated membrane pore formation.

**TABLE 1:** Major activation pathways and molecular regulators of pyroptosis.

Pyroptosis pathway	Primary sensors/Triggers	Key caspases	Gasdermin involved	Core molecular features	Representative biological contexts
Canonical inflammasome pathway	NLRP3, AIM2, NLRC4 sensing PAMPs/DAMPs	Caspase-1	GSDMD	Inflammasome assembly → caspase-1 activation → GSDMD cleavage → IL-1β/IL-18 maturation and release	Infection, atherosclerosis, IBD, autoimmune diseases
Non-canonical inflammasome pathway	Cytosolic LPS	Caspase-4/5 (human), caspase-11 (mouse)	GSDMD	Direct LPS binding to caspases → GSDMD cleavage → secondary NLRP3 activation	Gram-negative infection, sepsis
Apoptosis-associated pyroptosis	Chemotherapy, mitochondrial stress	Caspase-3	GSDME	Conversion of apoptosis to inflammatory cell death via GSDME cleavage	Cancer therapy, drug-induced tumor cell death
Caspase-8-mediated pyroptosis	Death receptor signaling, microbial infection	Caspase-8	GSDMD/GSDMC	Links extrinsic apoptosis to pyroptosis; involved in PANoptosis	Infection, sterile inflammation, cancer
Granzyme-mediated pyroptosis	Cytotoxic lymphocyte attack	Granzyme B	GSDME	Direct gasdermin cleavage independent of caspases	Tumor immune surveillance
Post-translational regulation	Ubiquitination, phosphorylation, succination	—	GSDMD/GSDME	Controls gasdermin stability, cleavage efficiency, and pore activity	Cancer, metabolic diseases
Non-coding RNA regulation	lncRNAs, circRNAs, miRNAs	Indirect	GSDMD/inflammasome components	Epigenetic fine-tuning of pyroptosis signaling	Diabetic nephropathy, cardiovascular disease
Ion flux-dependent amplification	K^+^ efflux, Ca^2+^ influx	Caspase-1	GSDMD	Ionic imbalance amplifies inflammasome activation and cell lysis	Acute inflammation

Schematic overview of the core molecular pathways underlying pyroptosis. Pyroptosis is initiated through canonical inflammasome activation, non-canonical caspase pathways, and apoptosis-associated caspase signaling, all converging on gasdermin (GSDM) cleavage and membrane pore formation. The release of pro-inflammatory cytokines and damage-associated molecular patterns (DAMPs) amplifies inflammatory responses and links pyroptosis to innate immunity and disease pathogenesis.

### 2.2 The role of pyroptosis in tumor occurrence and development

#### 2.2.1 Effects of pyroptosis on tumor cell growth and apoptosis

Pyroptosis, a form of programmed inflammatory cell death mediated primarily by the GSDM family, has emerged as a critical regulator of tumor cell fate, influencing both tumor growth inhibition and the TME. The molecular mechanism underlying pyroptosis-induced tumor cell death typically involves activation of inflammasomes, leading to caspase cleavage (notably caspase-1, caspase-3, caspase-8, and caspase-9) and subsequent cleavage of GSDM proteins such as GSDMD and GSDME. The GSDMD-Ns of these GSDMs oligomerize to form pores in the plasma membrane, causing cell swelling, membrane rupture, and release of pro-inflammatory cytokines like IL-1β and IL-18, which further amplify anti-tumor immune responses (Ju et al., 2021; Wen et al., 2023; Tan et al., 2021). Various studies have demonstrated that inducing pyroptosis in tumor cells leads to inhibition of tumor proliferation, migration, and invasion across multiple cancer types including esophageal cancer, lung cancer, hepatocellular carcinoma (HCC), and anaplastic thyroid carcinoma. For instance, inhibition of endogenous hydrogen sulfide in esophageal cancer cells promoted pyroptosis via reactive oxygen species (ROS)-mediated NF-κB signaling, resulting in suppressed tumor growth both *in vitro* and *in vivo* (Wang H. G. et al., 2023). Similarly, natural compounds such as isoquercitrin and osthole have been shown to induce pyroptosis through activation of the NLRP3 inflammasome and caspase-3/GSDME pathways, respectively, thereby inhibiting lung cancer and cervical cancer cell growth (Fan et al., 2025; Wang J. et al., 2022). Phototherapy approaches using mitochondria-targeted cyanine dyes or carbon dot photosensitizers have also been optimized to trigger pyroptosis alongside apoptosis, enhancing antitumor efficacy and immune activation (Zhao X. et al., 2024; Chen T. et al., 2024). Mechanistically, pyroptosis not only directly kills tumor cells but also remodels the TIME by recruiting and activating DCs and CTLs, converting immunologically “cold” tumors into “hot” tumors that are more susceptible to immunotherapy (Inkol et al., 2024; Li M. et al., 2023). Furthermore, pyroptosis induction has been linked to overcoming apoptosis resistance, a common hurdle in cancer therapy, by providing an alternative cell death pathway that amplifies treatment-induced tumor cell killing (Li M. et al., 2023; Zhou et al., 2025). However, tumor cells have evolved sophisticated strategies to evade pyroptosis, which limits its therapeutic potential. One such mechanism involves the overexpression of Sorcin in HCC, which interacts with the NLRP3 inflammasome to inhibit pyroptosis, thereby promoting tumor proliferation and metastasis (Li Z. et al., 2023). Another example is the elevated expression of Niemann-Pick C1 (NPC1) in various cancers, which maintains cholesterol homeostasis to protect tumor cells from pyroptosis, and its inhibition sensitizes tumors to pyroptotic cell death (Zhang C. et al., 2025). Additionally, tumor cells may downregulate GSDM expression or interfere with caspase activation to suppress pyroptosis execution (Wang Y. et al., 2024; Ren Y. et al., 2023). Collectively, these findings underscore the dual role of pyroptosis in tumor biology: as a potent tumor suppressor mechanism through direct tumor cell killing and immune activation, and as a process that can be subverted by tumors to facilitate survival. Harnessing pyroptosis for cancer therapy thus requires a nuanced understanding of these molecular mechanisms to effectively induce pyroptosis in tumor cells while overcoming their evasion strategies.

#### 2.2.2 Interaction between pyroptosis and TIME

Pyroptosis serves as a critical mechanism in converting immunologically “cold” tumors, characterized by low immune cell infiltration and poor responsiveness to immunotherapy, into “hot” tumors with robust immune activation. This transformation is essential for improving the efficacy of immune checkpoint blockade (ICB) therapies, which rely on pre-existing antitumor immune responses. Pyroptosis-induced inflammation promotes the release of tumor-associated antigens (TAAs) and DAMPs, thereby enhancing DC maturation and T cell priming, which collectively increase tumor immunogenicity (Wang W. et al., 2022; Yang Y. et al., 2023). Experimental models have demonstrated that pyroptosis induction sensitizes tumors to anti-PD-1 therapy by increasing CD8^+^ T cell infiltration and reducing immunosuppressive cell populations such as myeloid-derived suppressor cells (MDSCs) and regulatory T cells (Tregs) (Wu J. et al., 2022; Wu F. et al., 2023). Nanoparticle-based delivery systems and small molecule agents that trigger pyroptosis have been developed to exploit this mechanism, successfully remodeling the TIME and enhancing immunotherapy responses in otherwise refractory tumors (Chen et al., 2024a; Liu et al., 2026a; Liu et al., 2026b).

Furthermore, pyroptosis influences the polarization and function of tumor-associated macrophages (TAMs), shifting them toward a pro-inflammatory M1 phenotype that supports antitumor immunity, while suppressing the immunosuppressive M2 phenotype (Huang X. et al., 2022; Ji et al., 2023). Dietary polyphenols and epigenetic modulators have been reported to induce pyroptosis and modulate TAM polarization, thereby reshaping the TIME to favor immune activation (Huang X. et al., 2022). In addition, pyroptosis-related gene signatures correlate with immune cell infiltration patterns, immune checkpoint expression, and patient prognosis across multiple cancer types, underscoring the integral role of pyroptosis in tumor-immune interactions (Li L. et al., 2024; Chen et al., 2021).

The pro-inflammatory environment generated by pyroptosis also modulates immune checkpoint expression, with observed upregulation of PD-L1 and other checkpoint molecules in response to pyroptosis-related signaling, suggesting a complex interplay between pyroptosis and immune evasion mechanisms (Zhang Y. et al., 2025; Yin Q. et al., 2024). Nonetheless, the net effect of pyroptosis-induced inflammation is the enhancement of tumor immunogenicity and promotion of effective antitumor immune responses, particularly when combined with ICB therapies (Wang W. et al., 2022).

One of the major challenges in cancer immunotherapy is the presence of “cold tumors,” which are characterized by low immune cell infiltration, minimal antigen presentation, and poor responsiveness to ICIs. Pyroptosis has emerged as a key mechanism capable of converting these immunologically inert tumors into “hot tumors” with active immune microenvironments (Li M. et al., 2023; Wang W. et al., 2022). The inflammatory nature of pyroptosis facilitates the release of TAAs, DAMPs, and pro-inflammatory cytokines, which collectively enhance DC activation and T cell priming, essential steps for effective antitumor immunity (Yang Y. et al., 2023; Zhou et al., 2022b).

Experimental studies have demonstrated that induction of pyroptosis via chemotherapeutic agents, nanomedicine, or genetic modulation increases infiltration of CD8^+^ T cells and NK cells into tumor sites, thereby enhancing immune surveillance and tumor cell killing (Yang et al., 2024; Li F. et al., 2023). For example, pyroptosis induction by histone deacetylase inhibitors in triple-negative breast cancer models resulted in increased CD8^+^ lymphocyte infiltration and GzmB expression, correlating with tumor growth inhibition (Yang et al., 2024). Similarly, vesicular stomatitis virus (VSV)-mediated pyroptosis sensitized immunologically cold tumors to checkpoint blockade by recruiting cytotoxic lymphocytes (Lin et al., 2022).

Moreover, pyroptosis modulates the TME by reducing immunosuppressive cell populations such as MDSCs and Tregs, and promoting the polarization of TAMs toward the M1 phenotype, which supports antitumor immunity (Wu F. et al., 2023; Ji et al., 2023). This remodeling of the immune landscape is critical for overcoming immune evasion and resistance to ICIs. Nanoparticle-based pyroptosis inducers have been engineered to specifically target tumor cells, inducing localized pyroptosis and enhancing immune cell infiltration without systemic toxicity (Chen et al., 2024a; Zhao L. et al., 2024).

Clinical and bioinformatic analyses further support the prognostic significance of pyroptosis-related gene signatures in predicting immune infiltration and response to immunotherapy across various cancers, including bladder cancer, gastric cancer, and gliomas (Chen et al., 2021; Zhou et al., 2022b; Cai Y. et al., 2022). These findings underscore the therapeutic potential of pyroptosis induction as a strategy to convert cold tumors into hot tumors, thereby improving the efficacy of immunotherapies.

#### 2.2.3 Heterogeneous roles of pyroptosis in different tumor types

Pyroptosis is increasingly recognized as a double-edged sword in cancer biology. While it can suppress tumor growth by directly eliminating malignant cells and stimulating antitumor immunity, excessive or persistent pyroptotic signaling may also promote tumor progression through chronic inflammation. This apparent contradiction suggests that the biological consequences of pyroptosis are highly context dependent rather than universally beneficial or detrimental.

On one hand, activation of pyroptosis results in the release of tumor antigens, DAMPs, and pro-inflammatory cytokines such as IL-1β and IL-18, thereby enhancing dendritic cell maturation, cytotoxic T-cell recruitment, and immune checkpoint blockade responsiveness (Fang et al., 2023). Several studies have demonstrated that pyroptosis-associated gene signatures correlate with increased immune infiltration and improved therapeutic outcomes in multiple cancers, supporting its role as an immunogenic form of cell death (Kuo et al., 2023). On the other hand, sustained pyroptotic activity may establish a pro-tumorigenic inflammatory microenvironment. The context-dependent nature of pyroptosis is particularly evident in gliomas (Sun et al., 2023). Multi-omics analyses have identified distinct pyroptosis-associated molecular subgroups with markedly different immune landscapes and clinical outcomes (Chen M. et al., 2024). Interestingly, tumors with high pyroptosis scores often exhibit increased immune infiltration but worse survival, suggesting that immune-cell abundance alone does not necessarily translate into effective antitumor immunity. Instead, the composition and functional state of infiltrating immune cells appear to be critical determinants of outcome (Zhou et al., 2026).

To capture this heterogeneity, several scoring systems, including the Pyroptosis Immune Prognostic Model (PIPM) and PyroScore, have been developed to integrate pyroptosis-related gene expression with immune characteristics (Shen et al., 2025). These models show promising prognostic and predictive value across multiple cancer types. However, most have been established using retrospective transcriptomic datasets and lack prospective clinical validation. Furthermore, substantial variability exists in the biomarkers and algorithms used, limiting their immediate clinical applicability (Gao et al., 2023; Zheng et al., 2022).

**FIGURE 2:**
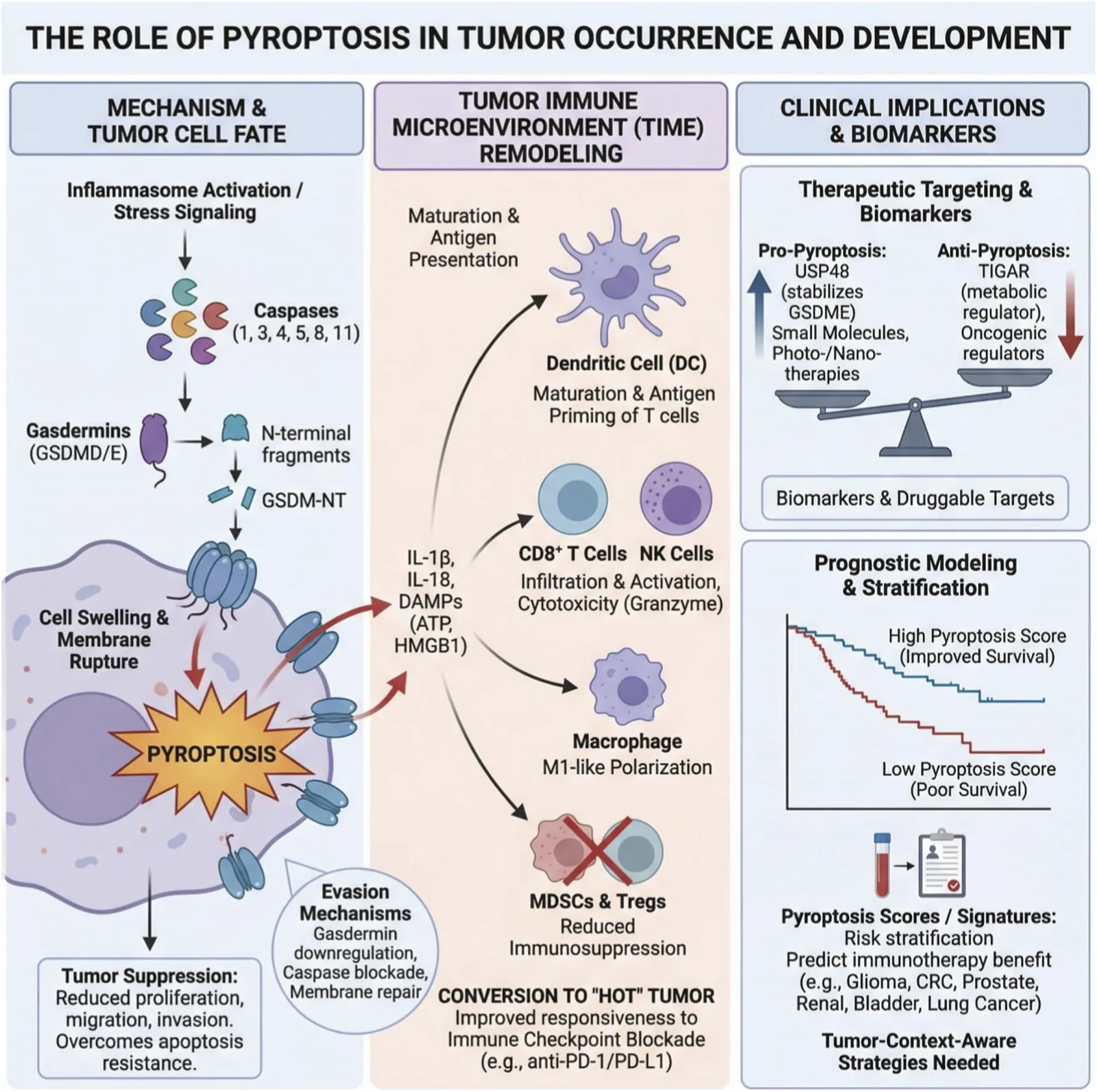
Context-dependent roles of pyroptosis in cancer.

In summary, the heterogeneous roles of pyroptosis in different tumor types reflect the complex interplay between tumor cells and the immune microenvironment. The development and validation of pyroptosis scoring systems provide valuable tools for clinical application, enabling improved prognostic prediction and personalized therapy, particularly in gliomas. Future research focusing on the molecular mechanisms underlying pyroptosis heterogeneity and its modulation of the TME will be pivotal for translating these insights into effective therapeutic interventions (Figure 2).

Dual roles of pyroptosis in tumor development and antitumor immunity. Pyroptosis can suppress tumor growth by inducing inflammatory tumor cell death and enhancing immune cell recruitment, while persistent or dysregulated pyroptosis may promote tumor progression by sustaining a pro-tumorigenic inflammatory microenvironment. These opposing effects highlight the context-dependent nature of pyroptosis in cancer.

### 2.3 Pyroptosis and inflammatory diseases and autoimmune diseases

#### 2.3.1 The mechanisms of pyroptosis in atherosclerosis

Endothelial cells (ECs) are among the first vascular cells affected by pyroptosis in atherosclerosis. NLRP3 inflammasome activation in ECs induces pyroptotic death, resulting in endothelial dysfunction, increased vascular permeability, and enhanced leukocyte adhesion, thereby promoting lesion initiation (Liu X. et al., 2023; Bai et al., 2021). Experimental models have demonstrated that pharmacological inhibition of NLRP3 or downstream effectors such as caspase-1 and GSDMD attenuates endothelial pyroptosis and reduces plaque formation, highlighting the therapeutic potential of targeting this pathway (Lin et al., 2021; Xing et al., 2020). Moreover, recent studies elucidate that ncRNAs, including miRNAs and lncRNAs, regulate NLRP3 inflammasome expression and activity in ECs, modulating pyroptosis and vascular inflammation (Wang Y. P. et al., 2025; Khojali et al., 2024). For instance, miR-302c-3p directly targets NLRP3 to suppress endothelial pyroptosis, while lncRNA HIF1A-AS2 delivered *via* endothelial extracellular vesicles promotes pyroptosis by upregulating NLRP3 (Bai et al., 2021; Li P. et al., 2023). Additionally, upstream signaling pathways such as NF-κB and thioredoxin-interacting protein (TXNIP) modulate NLRP3 inflammasome priming and activation in ECs, linking oxidative stress and inflammation to pyroptosis (Hendawy et al., 2025; Li J. et al., 2022). The involvement of pyroptosis in ECs is further supported by *in vivo* evidence showing that interventions like pulsed electromagnetic fields and natural compounds (e.g., salidroside) reduce endothelial pyroptosis and ameliorate atherosclerotic lesions by inhibiting NLRP3 inflammasome activation (Xing et al., 2020; Cheng et al., 2025). Collectively, these findings underscore that NLRP3 inflammasome-mediated pyroptosis in vascular ECs is a critical mechanism driving endothelial dysfunction and atherosclerosis progression, offering promising targets for therapeutic intervention.

Moreover, pyroptotic ECs release microparticles containing inflammatory mediators, which propagate vascular inflammation and facilitate crosstalk among ECs, macrophages, and smooth muscle cells, accelerating atherogenesis (Sun et al., 2021). The amplification of inflammation by pyroptosis is tightly regulated by multiple signaling pathways, including NF-κB, TXNIP, and JAK/STAT, which modulate inflammasome activation and cytokine production (Hendawy et al., 2025; Li J. et al., 2022; Zhou and Song, 2023). Therapeutic interventions targeting pyroptosis-related molecules have demonstrated efficacy in reducing inflammatory cytokine release and stabilizing plaques. For instance, bone marrow mesenchymal stem cell-derived exosomes (MSC-Exos) suppress NLRP3/caspase-1/GSDMD-mediated pyroptosis, decreasing IL-1β and IL-18 levels and alleviating atherosclerosis progression (Bai et al., 2023). Beyond MSC-derived exosomes, increasing evidence indicates that extracellular vesicles (EVs) from diverse biological sources also possess significant anti-inflammatory and anti-pyroptotic properties (Khalilzad et al., 2025a). Placenta-derived EVs, particularly those isolated from human placental mesenchymal stromal cells and trophoblast-associated tissues, exhibit potent immunomodulatory effects through suppression of NLRP3 inflammasome activation, reduction of pro-inflammatory cytokine production, and promotion of tissue repair (Ruan et al., 2025; Khalilzad et al., 2025b). Their favorable safety profile and naturally immunosuppressive characteristics make them attractive candidates for inflammatory disease treatment.

#### 2.3.2 Regulatory network of pyroptosis in DN

The regulatory network of pyroptosis in DN is complex and involves multiple molecular players, including ncRNAs, inflammasomes, and signaling pathways that converge to exacerbate renal cell injury and inflammation. Recent studies have highlighted the critical role of ncRNAs, including lncRNAs and circRNAs, in modulating pyroptosis and thereby influencing the progression of DN. For instance, lncRNA MALAT1 is significantly upregulated in DN patients and correlates with increased serum caspase-1 and IL-18 levels, markers of pyroptosis, indicating its involvement in promoting renal tubular epithelial cell injury through pyroptotic mechanisms (Shoeib et al., 2023). Similarly, circ_0004951 promotes pyroptosis in renal tubular epithelial cells by sponging miR-93-5p, which normally suppresses NLRP3 inflammasome activation; thus, circ_0004951 upregulation leads to enhanced NLRP3-mediated pyroptosis and inflammation in DN (Wang Y. et al., 2022). These ncRNAs act as upstream regulators that fine-tune inflammasome activation and pyroptotic cell death, underscoring their potential as biomarkers and therapeutic targets.

Inflammasome inhibitors have garnered attention as potential therapeutic agents in DN. Natural compounds such as puerarin, syringaresinol, and tanshinone IIA have demonstrated efficacy in mitigating pyroptosis by inhibiting caspase-1 activation and NLRP3 inflammasome assembly, thereby reducing inflammatory cytokine release and renal damage (Chen Q. et al., 2024; Li G. et al., 2023; Wu Q. et al., 2023). Furthermore, hirudin ameliorates DN by suppressing GSDMD-mediated pyroptosis through regulation of interferon regulatory factor 2 (IRF2), highlighting the role of transcriptional regulation in pyroptosis control (Han et al., 2023). The identification of pyroptosis-related genes and their associated pathways has facilitated the screening of numerous candidate drugs that target key nodes in the pyroptosis network, offering a promising avenue for DN treatment (Yan et al., 2023).

The NLRP3 inflammasome is a critical driver of pyroptosis in DN, and its inhibition has emerged as a promising therapeutic strategy to attenuate renal inflammation and fibrosis. Multiple pharmacological agents and natural compounds have demonstrated efficacy in suppressing inflammasome activation and downstream pyroptotic pathways in preclinical models of DN. For example, puerarin administration in streptozotocin-induced diabetic mice reduces fasting blood glucose, alleviates glomerular lesions, and inhibits caspase-1-mediated pyroptosis in podocytes, as evidenced by decreased IL-1β and IL-18 release (Chen Q. et al., 2024). Similarly, syringaresinol exerts renoprotective effects by activating NRF2 signaling, which suppresses NLRP3/caspase-1/GSDMD-mediated pyroptosis and oxidative stress in diabetic mice (Li G. et al., 2023). Tanshinone IIA, derived from Salvia miltiorrhiza, inhibits oxidative stress-induced pyroptosis in glomerular by modulating the TXNIP/NLRP3 inflammasome axis, thereby mitigating renal injury (Wu Q. et al., 2023). These findings highlight the therapeutic potential of targeting upstream oxidative stress and inflammasome activation to prevent pyroptotic cell death.

Other agents such as hirudin inhibit GSDMD-mediated pyroptosis by downregulating interferon regulatory factor 2, resulting in improved renal function and reduced tubular injury in diabetic models (Han et al., 2023). Additionally, the MIF inhibitor Chicago sky blue 6B suppresses NLRP3 inflammasome activation and fibrosis by downregulating the NLRP3/caspase-1/GSDMD/IL-1β pathway in DN (Chen et al., 2026). The identification of pyroptosis-related gene signatures has facilitated the screening of 65 potential therapeutic compounds targeting key pyroptosis mediators, providing a rich resource for drug development (Yan et al., 2023).

#### 2.3.3 Relationship between pyroptosis and autoimmune diseases

Pyroptosis, a highly inflammatory form of programmed cell death mediated by GSDM family proteins, has been increasingly recognized as a pivotal mechanism contributing to the pathogenesis of various autoimmune diseases, including RA, systemic lupus erythematosus (SLE), and multiple sclerosis (MS). In RA, excessive pyroptosis is driven by inflammatory mediators such as tumor necrosis factor (TNF) and activation of the NLRP3 inflammasome, which exacerbate synovial inflammation and joint damage. Fibroblast-like synoviocytes (FLSs) in RA patients demonstrate elevated pyroptotic activity, with increased expression of caspase-1, GSDMD, and pro-inflammatory cytokines IL-1β and IL-18, contributing to chronic synovitis and cartilage degradation (Song et al., 2024; Xin et al., 2024; Ren C. et al., 2023). Similarly, in SLE, pyroptosis facilitates the release of nuclear antigens and pro-inflammatory cytokines, perpetuating autoantibody production and systemic inflammation. Elevated pyroptosis markers have been detected in immune cells and affected tissues, highlighting its role in disease progression (Song et al., 2024; Yao et al., 2024). In MS, pyroptosis of central nervous system cells, including oligodendrocytes, is implicated in demyelination and neuroinflammation. Activation of caspase-11 and the NLRP3 inflammasome promotes pyroptotic cell death, which amplifies immune cell infiltration and tissue injury (Song et al., 2024; Song et al., 2022). The overactivation of pyroptosis leads to sustained chronic inflammation and tissue damage, characteristic of autoimmune pathology. Mechanistically, inflammasome activation triggers caspase-1 or caspase-11/4/5 cleavage of GSDMs, forming membrane pores that cause cell lysis and release of inflammatory mediators, thereby disrupting immune homeostasis and promoting autoimmunity (Burdette et al., 2021; Wu et al., 2021a). Therapeutic strategies targeting pyroptosis pathways, including inhibition of NLRP3 inflammasome, caspases, or GSDM pore formation, have shown promise in preclinical models of autoimmune diseases. For example, nanoliposomes responsive to pyroptotic signals have been developed to deliver anti-pyroptotic drugs selectively to inflamed tissues, effectively reducing autoimmune inflammatory damage in arthritis and inflammatory bowel disease (IBD) models (Xu et al., 2024; Pan et al., 2024). Moreover, agents such as dimethyl fumarate and disulfiram have demonstrated the ability to inhibit GSDMD-mediated pyroptosis, thereby alleviating inflammation in autoimmune conditions (Bandharam et al., 2023; Gong W. et al., 2024). Collectively, these findings underscore the critical role of pyroptosis in autoimmune disease pathogenesis, where its dysregulation drives chronic inflammation and tissue injury. The excessive activation of pyroptosis not only amplifies inflammatory cascades but also promotes the recruitment and activation of autoreactive immune cells, perpetuating a vicious cycle of immune-mediated damage. Understanding the molecular mechanisms linking pyroptosis to autoimmunity provides valuable insights for developing targeted therapies aimed at modulating pyroptotic pathways to restore immune tolerance and prevent tissue destruction in autoimmune diseases (Song et al., 2024; You et al., 2022; Zhang et al., 2021a). Additionally, environmental exposure has recently emerged as an important upstream regulator of pyroptosis and is increasingly recognized as a contributor to multiple chronic diseases. A growing body of evidence indicates that diverse environmental pollutants—including particulate matter, heavy metals, pesticides, engineered nanoparticles, per- and polyfluoroalkyl substances (PFAS), and microplastics—can activate pyroptotic signaling pathways in various tissues (Berkel and Cacan, 2023).

#### 2.3.4 Role of pyroptosis regulation in chronic intestinal diseases

Pyroptosis is increasingly recognized as an important mechanism in chronic intestinal diseases, particularly inflammatory bowel disease (IBD), including ulcerative colitis and Crohn’s disease (Zhang et al., 2022a). In response to microbial infection, intestinal dysbiosis, and endogenous danger signals, the NLRP3 inflammasome activates caspase-1, which subsequently cleaves GSDMD and promotes the release of IL-1β and IL-18 (Shao et al., 2019). This NLRP3–caspase-1–GSDMD axis disrupts intestinal epithelial barrier integrity, amplifies mucosal inflammation, and contributes to persistent immune dysregulation in IBD. Consistently, reviews focused on IBD have summarized that NLRP3 inflammasome activation and pyroptosis are closely associated with intestinal inflammation and disease progression.

The relationship between gut microbiota and pyroptosis further strengthens this pathogenic axis (Pérez-Rodríguez and Mercanoglu Taban, 2019). Dysbiosis may promote excessive inflammasome activation in intestinal epithelial cells and macrophages, whereas microbiota-derived metabolites, particularly short-chain fatty acids such as butyrate, help maintain epithelial homeostasis and regulate intestinal immune responses (Shao et al., 2019; Fingelkurts and Fingelkurts, 2019). Therefore, targeting the microbiota–NLRP3–caspase-1–GSDMD pathway, either by inflammasome inhibition or microbiota-modulating strategies, may provide a promising therapeutic direction for IBD and related chronic intestinal inflammatory disorders (Zhang et al., 2022a). Therapeutically, inhibition of the NLRP3–caspase-1–GSDMD pathway, together with microbiota-modulating approaches, including extracellular vesicles, microbial metabolites, and emerging nanomedicine platforms, has demonstrated encouraging efficacy in preclinical models. Collectively, these findings identify the microbiota–inflammasome–pyroptosis axis as a central mechanism linking microbial dysbiosis, immune dysregulation, and epithelial barrier dysfunction in chronic intestinal diseases (Pérez-Rodríguez and Mercanoglu Taban, 2019).

**FIGURE 3:**
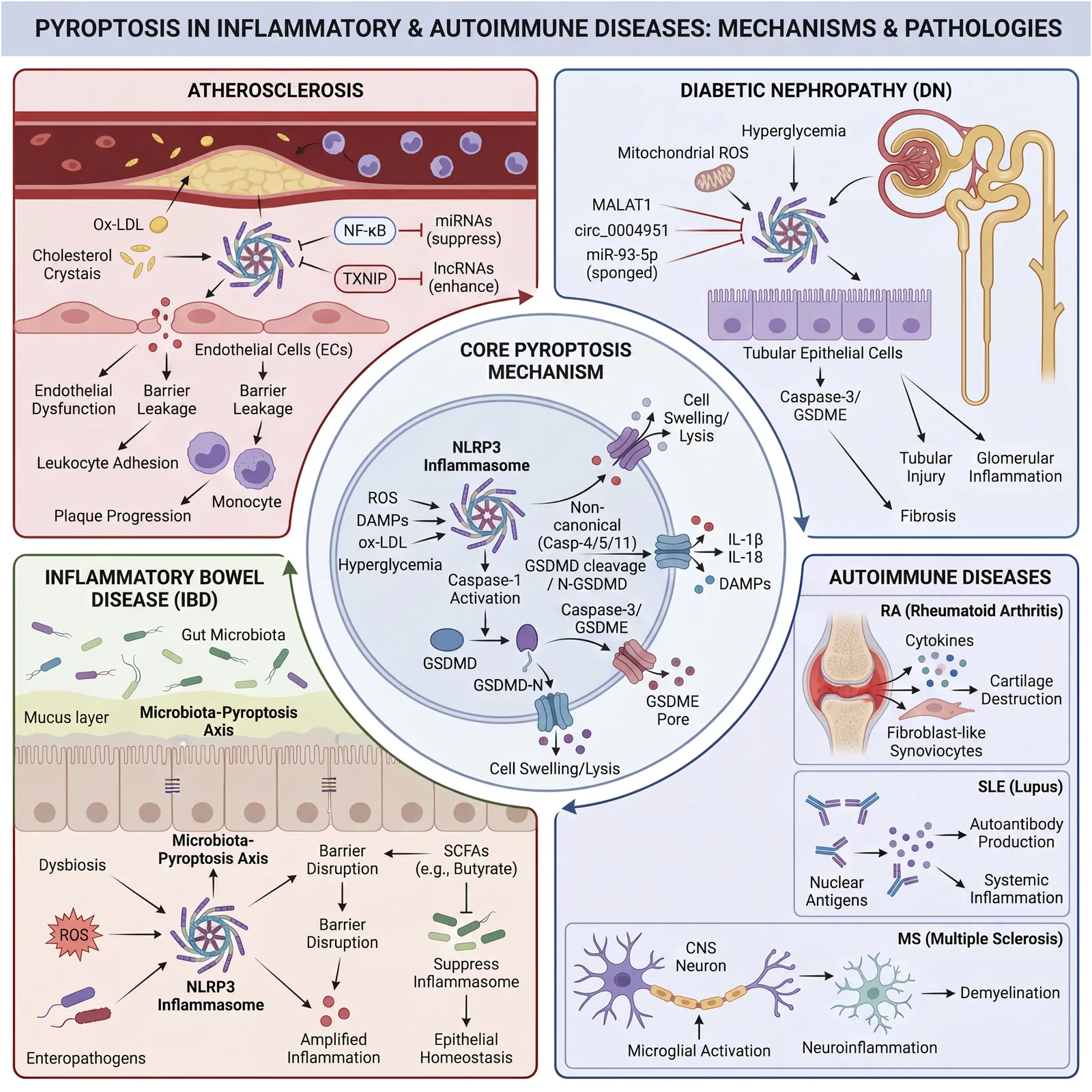
Pyroptosis in inflammatory, metabolic, and autoimmune diseases.

In summary, pyroptosis serves as a critical nexus in chronic intestinal diseases by mediating inflammatory cell death that disrupts epithelial barriers and modulates immune responses. Its regulation is intricately linked with gut microbiota dynamics, creating a complex network that influences disease pathogenesis and therapeutic outcomes. Advances in understanding pyroptosis mechanisms and their interactions with the microbiome have paved the way for novel therapeutic interventions, including small molecule inhibitors, biologics, natural products, stem cell-derived exosomes, and microbiota-targeted therapies. Future research focusing on the precise modulation of pyroptosis and microbiota balance holds promise for improving treatment efficacy and patient quality of life in chronic intestinal disorders (Figure 3).

Pathological involvement of pyroptosis across inflammatory, metabolic, and autoimmune disorders. Aberrant activation of inflammasomes and GSDM-mediated pyroptosis contributes to endothelial dysfunction, tissue injury, and chronic inflammation in diseases such as atherosclerosis, diabetic nephropathy, IBD, and autoimmune conditions.

### 2.4 Research progress on treatment strategies related to thermal death

Current therapeutic strategies targeting pyroptosis across different diseases are systematically summarized in Table 2, emphasizing both their translational potential and inherent limitations.

**TABLE 2:** Therapeutic strategies targeting pyroptosis: mechanisms, applications, and challenges.

Therapeutic strategy	Target level	Mechanism of action	Disease context	Therapeutic advantage	Key limitations
NLRP3 inflammasome inhibitors	Inflammasome assembly	Block sensor–ASC–caspase-1 complex formation	Atherosclerosis, IBD, autoimmune diseases	Strong anti-inflammatory efficacy	Infection susceptibility, long-term safety
Caspase-1 inhibitors	Execution initiation	Prevent cytokine maturation and gasdermin cleavage	Sepsis, metabolic inflammation	Rapid inflammation control	Limited disease specificity
Gasdermin pore inhibitors	Terminal execution	Block membrane pore formation	Autoimmune diseases, IBD	Direct suppression of tissue damage	May impair host defense
Pyroptosis inducers (small molecules)	Gasdermin activation	Trigger inflammatory tumor cell death	Cancer	Converts “cold” tumors to “hot”	Risk of systemic inflammation
Chemotherapy-induced GSDME activation	Caspase-3–GSDME axis	Apoptosis-to-pyroptosis switch	Solid tumors	Enhances immunogenic cell death	Tumor heterogeneity
Nanomaterial-based delivery systems	Spatial regulation	Tumor- or tissue-specific pyroptosis modulation	Cancer, inflammatory diseases	Precision targeting, reduced toxicity	Manufacturing complexity
Stem cell-derived exosomes	Signaling modulation	Suppress excessive pyroptosis	Cardiovascular, renal diseases	Excellent biocompatibility	Standardization challenges
RNA-based therapeutics	Epigenetic regulation	Target pyroptosis-related ncRNAs	Metabolic and autoimmune diseases	High specificity	Delivery efficiency
Combination therapy (ICB + pyroptosis)	Immune synergy	Amplify antitumor immunity	Cancer immunotherapy	Improved response rate	Immune-related adverse events

#### 2.4.1 Small molecule inhibitors and activators

The development of small molecule modulators targeting the NLRP3 inflammasome and caspase family members represents a pivotal strategy in regulating pyroptosis, with significant therapeutic implications across diverse diseases, including inflammatory disorders and cancer. Small molecule inhibitors of caspase-1, such as NSC697923, have demonstrated potent suppression of enzymatic activity with an IC50 of 1.737 μM, effectively inhibiting NLRP3 inflammasome activation and downstream pyroptotic events in macrophages stimulated by various danger signals (Cao et al., 2022). Mechanistically, NSC697923 not only impedes caspase-1 enzymatic function but also downregulates NLRP3 expression by suppressing NF-κB signaling and disrupting the interaction between RIP2 and pro-caspase-1, thereby blocking inflammasome priming. This dual action culminates in reduced GSDMD cleavage and pyroptosis, which translates into alleviation of inflammation *in vivo*, as evidenced in a gouty arthritis model. Similarly, manoalide, a selective small molecule inhibitor of NLRP3, acts downstream of potassium and chloride efflux as well as mitochondrial dysfunction, covalently binding to Lys377 of NLRP3 to block its interaction with NEK7, thereby inhibiting inflammasome assembly and ameliorating autoimmune encephalomyelitis in mice (Li et al., 2022c). These findings underscore the therapeutic potential of targeting inflammasome components with small molecules to modulate pyroptosis and inflammatory responses.

In the context of cancer therapy, pyroptosis induction via small molecules has emerged as a promising approach to enhance antitumor immunity. For example, the small molecule stabilizer of the MYC G-quadruplex, D089, induces endoplasmic reticulum stress in multiple myeloma cells, triggering both cellular senescence and pyroptosis through caspase-1 activation, GSDMD cleavage, and inflammasome assembly (Gaikwad et al., 2020). The pyroptotic cell death elicited by D089 is characterized by morphological changes distinct from apoptosis, such as cell swelling and membrane rupture, and is accompanied by the release of pro-inflammatory cytokines and DAMPs, which potentiate immune responses against the tumor. Furthermore, engineered nanoplatforms co-delivering small molecule inhibitors targeting PI3K/mTOR and CDK pathways have been designed to induce GSDME-mediated immunogenic pyroptosis in tumor cells, thereby reprogramming the immunosuppressive TME and enhancing the efficacy of ICB therapies (Yang Q. et al., 2022). These nanomicelles exploit the TME’s high glutathione levels for selective activation and deep tumor penetration, illustrating the sophisticated design of small molecule delivery systems to maximize pyroptosis induction and antitumor immunity.

From a translational perspective, several pyroptosis-related inhibitors have already progressed beyond preclinical evaluation. VX-765, an orally bioavailable caspase-1 inhibitor, has been evaluated in clinical studies for epilepsy and inflammatory disorders, demonstrating favorable tolerability and anti-inflammatory activity. In addition, IL-1β-targeting therapies, such as canakinumab and anakinra, have achieved regulatory approval for inflammatory diseases and indirectly validate the therapeutic value of inflammasome-associated pathways in humans (Ridker et al., 2017a). Notably, secondary analyses of the CANTOS trial revealed a reduction in lung cancer incidence and mortality among patients receiving canakinumab, suggesting that modulation of pyroptosis-associated inflammatory signaling may have broader clinical implications beyond inflammatory disorders (Ridker et al., 2017b).

#### 2.4.2 Nanomaterial-mediated regulation of pyroptosis

Nanomaterials have emerged as powerful tools in the precise modulation of pyroptosis, offering innovative strategies for disease treatment, particularly in cancer and inflammatory disorders. Their unique physicochemical properties—such as size, shape, surface charge, and chemical composition—enable them to interact specifically with cellular components involved in pyroptosis pathways, thereby allowing targeted and controlled induction or inhibition of this form of programmed cell death (Yu et al., 2025). One of the critical advantages of nanomaterials is their ability to serve as carriers for pyroptosis-regulating molecules, ensuring precise delivery to diseased tissues while minimizing systemic toxicity. For example, calcium-based nanomaterials have been engineered to induce calcium overload in tumor cells, disrupting mitochondrial calcium homeostasis and activating caspase-1-mediated cleavage of GSDMD, which triggers pyroptosis (Li et al., 2025b). Such nanomodulators can be coated with targeting ligands like hyaluronic acid to enhance tumor specificity and exploit the acidic TME for controlled drug release, thereby improving therapeutic outcomes.

Moreover, nanomaterials can be designed to generate ROS within tumor cells, a key upstream signal in pyroptosis activation. Vanadium-based biodegradable nanoparticles exemplify this approach by catalyzing reactions that elevate intracellular ROS, activating inflammasomes such as NLRP3, and promoting pyroptotic cell death alongside pyroptosis, thereby amplifying antitumor effects (Zhang J. et al., 2025). Similarly, nanozymes with enzyme-mimicking catalytic activities can modulate ROS levels and inflammasome activation, inducing pyroptosis and other regulated cell death pathways to enhance cancer therapy (Zhang Y. et al., 2023). These multifunctional nanomaterials also facilitate combination therapies, integrating photodynamic, photothermal, and chemodynamic modalities to synergistically induce pyroptosis and potentiate immune responses.

In addition to direct induction, nanomaterials can modulate the TIME by promoting pyroptosis-mediated release of pro-inflammatory cytokines and DAMPs, which stimulate DC maturation and cytotoxic T cell infiltration. This immunogenic cell death enhances antitumor immunity and improves the efficacy of ICB therapies (Xie et al., 2025; Ge et al., 2024). For instance, platelet-camouflaged nanovehicles have been developed to deliver pyroptosis-inducing agents specifically to bladder cancer cells, triggering caspase-3/GSDME-dependent pyroptosis and remodeling the immunosuppressive microenvironment (Tian et al., 2024). Likewise, nanospiky copper peroxide nanoparticles regulate lactate metabolism and induce both cuproptosis and pyroptosis, further enhancing antigen presentation and antitumor immune responses (Liu B. et al., 2025).

Despite these promising advances, challenges remain in balancing the beneficial and adverse effects of nanomaterial-induced pyroptosis. Overactivation may lead to excessive inflammation or damage to normal tissues, while inadequate targeting can reduce therapeutic efficacy. Strategies such as surface modifications, controlled activation mechanisms, and the use of mRNA-based nanomedicines are being explored to achieve intelligent and precise pyroptosis modulation tailored to the dynamic disease microenvironment (Yu et al., 2025). Furthermore, nanomaterials facilitate the co-delivery of multiple agents, such as epigenetic modulators and inflammasome activators, to enhance pyroptosis by upregulating GSDM expression and inflammasome activation, thereby overcoming tumor resistance and improving immunotherapy outcomes (Niu et al., 2022).

Nanocarriers provide a highly efficient platform for the targeted delivery of pyroptosis modulators, overcoming limitations of conventional small-molecule drugs such as poor solubility, rapid degradation, and off-target toxicity. Various nanocarriers—including liposomes, polymeric nanoparticles, metal-based nanomaterials, and biomimetic vehicles—have been engineered to transport pyroptosis-inducing agents directly to diseased cells. For example, calcium-based nanoparticles have been synthesized via microemulsion methods to deliver calcium ions that induce caspase-1/GSDMD-mediated pyroptosis in tumor cells, simultaneously enhancing antigen presentation and modulating the immunosuppressive TME (Li et al., 2026). Similarly, nanocarriers loaded with nigericin and decitabine have been developed to upregulate GSDMD expression and activate inflammasomes, effectively inducing pyroptosis and systemic antitumor immunity (Niu et al., 2022).

Recent advances in microfluidic technologies have provided powerful tools for the development and optimization of pyroptosis-targeted therapeutics. Microfluidic platforms enable precise control over nanoparticle synthesis, drug encapsulation, and formulation parameters, resulting in improved reproducibility, particle uniformity, and drug loading efficiency. Furthermore, microfluidic tumor-on-chip and organ-on-chip systems facilitate real-time evaluation of pyroptosis-inducing agents under physiologically relevant conditions, accelerating therapeutic screening and personalized treatment development (Yang et al., 2026). Nanoparticle-mediated delivery of immune checkpoint inhibitors has emerged as a promising strategy to enhance pyroptosis-associated antitumor immunity. Conventional systemic administration of anti-PD-1, anti-PD-L1, or anti-CTLA-4 antibodies is often limited by poor tumor accumulation and immune-related adverse events (Ghaneialvar et al., 2025). Advanced nanoparticle platforms improve intratumoral delivery, prolong circulation time, and reduce off-target toxicity (Li F. et al., 2023).

#### 2.4.3 Regulation of pyroptosis by stem cell-derived exosomes

MSC-Exos have emerged as potent modulators of pyroptosis, a form of inflammatory programmed cell death, thereby offering promising therapeutic avenues for mitigating tissue damage across various diseases. MSC-Exos exert their anti-pyroptotic effects through multiple signaling pathways and molecular cargos, including miRNAs, circRNAs, and proteins, which collectively regulate the activation of inflammasomes and downstream pyroptotic effectors. For instance, in intervertebral disc degeneration (IVDD), MSC-Exos inhibit NLRP3 inflammasome-mediated pyroptosis in nucleus pulposus cells by delivering miR-410, which directly targets NLRP3 mRNA, thus reducing caspase-1 activation and inflammatory cytokine release (Zhang et al., 2020). Similarly, human umbilical cord MSC-derived exosomes (UMSC-Exo) release circHIPK3, which downregulates miR-421 and upregulates FOXO3a, suppressing NLRP3 inflammasome activation and consequent pyroptosis in ischemic skeletal muscle injury models (Yan et al., 2020). In acute lung injury (ALI, MSC-Exos attenuate alveolar macrophage pyroptosis by delivering miRNAs that target caspase-1 and immunoregulatory proteins, thereby reducing inflammation and improving lung function (Liu P. et al., 2024). Moreover, dental follicle stem cell-derived exosomes (DFSC-Exos) have been shown to inhibit NLRP3-mediated pyroptosis in periodontal ligament cells via miR-140-3p, which modulates the DNMT1/SOCS1/NF-κB axis, ultimately reducing osteoclastogenesis and root resorption (Li et al., 2025c). In diabetic wound healing, hair follicle MSC-derived exosomal long non-coding RNA H19 suppresses NLRP3 inflammasome activation, promoting keratinocyte proliferation and migration while inhibiting pyroptosis (Yang H. et al., 2023). Furthermore, embryonic stem cell-derived exosomes (ES-Exos) mitigate doxorubicin-induced muscle toxicity by reducing NLRP3 inflammasome components and shifting macrophage polarization from pro-inflammatory M1 to anti-inflammatory M2 phenotypes, thereby attenuating pyroptosis and fibrosis (Dessouki et al., 2020). Collectively, these studies demonstrate that MSC-Exos regulate pyroptosis through diverse molecular mechanisms, including miRNA-mediated suppression of inflammasome components, modulation of signaling pathways such as NF-κB, and alteration of macrophage polarization, leading to reduced inflammatory cell death and enhanced tissue repair.

The clinical translation of exosome-based drug delivery systems holds substantial promise due to their inherent biocompatibility, low immunogenicity, and ability to cross biological barriers. MSC-Exos can be engineered to carry specific therapeutic cargos, such as miRNAs or proteins, targeting pyroptosis-related pathways to enhance efficacy and specificity. For example, exosomes modified to overexpress miR-182-5p or miR-23b-3p have shown enhanced cardioprotective effects by targeting GSDMD and NEK7, respectively, thereby inhibiting pyroptosis in myocardial ischemia/reperfusion injury and experimental autoimmune encephalomyelitis models (Yue et al., 2022; Wang J. et al., 2023). Additionally, surface modification strategies, such as PEGylation and aptamer conjugation, have been employed to improve exosome circulation time and target specificity, as demonstrated in β-cell-targeting MSC-Exos that suppress NRF2-mediated pyroptosis to improve β-cell function in diabetes (Xia et al., 2024). Despite these advances, challenges remain in large-scale exosome production, standardized isolation methods, and ensuring consistent cargo loading. Furthermore, the heterogeneity of exosome populations and potential off-target effects necessitate rigorous preclinical and clinical evaluation. Nonetheless, the multifunctionality of MSC-Exos, combined with their capacity for precise modulation of pyroptosis, positions them as a versatile platform for developing novel therapies for inflammatory and degenerative diseases. Ongoing research focusing on optimizing exosome engineering, understanding biodistribution, and elucidating long-term safety profiles will be critical to realizing their full clinical potential.

#### 2.4.4 Gene editing and RNA interference technologies

Gene editing and RNA interference (RNAi) technologies have emerged as powerful tools to modulate pyroptosis-related gene expression, offering promising therapeutic strategies for various diseases characterized by dysregulated pyroptosis. Among these, CRISPR-Cas9 technology stands out as a precise and efficient method for targeted gene modification, enabling the knockout or activation of pyroptosis-associated genes to regulate inflammatory cell death pathways. For instance, in psoriasis, CRISPR-Cas9-mediated knockout of CASP1 and CASP5 in keratinocytes delivered via lipid nanoparticles effectively alleviated skin inflammation by suppressing pyroptosis, highlighting the therapeutic potential of gene editing in inflammatory skin disorders (Xu G. et al., 2025). Similarly, in cancer cells, CRISPR-Cas9 gene editing of caspase-8 demonstrated that caspase-8 accumulation and activation are critical for enhancing apoptosis and pyroptosis during combined hyperthermia and cisplatin therapy, suggesting that modulation of caspase-8 expression via gene editing can sensitize tumor cells to cell death (Zi et al., 2024). Moreover, CRISPR-Cas9 gene-edited macrophages lacking caspase-4 or caspase-1 revealed distinct roles in GSDMD cleavage and IL-1β secretion, underscoring the utility of gene editing to dissect pyroptosis mechanisms and identify therapeutic targets (Wang S. et al., 2022). Advancements in base editing technologies, such as bioorthogonally activatable cytosine base editors, enable on-demand and cell-type-specific activation of pyroptosis by inducing truncated GSDME expression, providing a novel approach for precise control of pyroptotic cell death (Ngai et al., 2022).

In addition to gene editing, RNAi and ncRNA-based therapies have been extensively explored to target pyroptosis-related pathways. RNA-binding proteins (RBPs) associated with pyroptosis have been implicated in acute myeloid leukemia prognosis, where risk models based on pyroptosis-related RBPs inform therapeutic strategies (Bin et al., 2022). lncRNAs and miRNAs modulate pyroptosis by regulating key inflammasome components and GSDM proteins. For example, in DN, silencing of lncRNA KCNQ1OT1 reduced oxidative stress and pyroptosis in renal tubular epithelial cells by upregulating miR-506-3p, illustrating the therapeutic potential of targeting ncRNA networks (Zhu et al., 2020). In UC, pericentriolar material 1 (PCM1) was found to promote NLRP3 inflammasome activation and GSDMD-mediated macrophage pyroptosis, implicating RNAi strategies to modulate PCM1 expression as a novel intervention (Niu et al., 2024). Furthermore, lncMCL1 was identified as a critical regulator of neural pyroptosis in epilepsy by stabilizing DDX3X protein through enhanced ubiquitination, suggesting that RNA-based modulation of lncRNAs can attenuate neuroinflammation (Wang H. et al., 2026).

The integration of CRISPR-Cas9 gene editing with RNAi approaches has also been applied in cancer immunotherapy. A nano-CRISPR scaffold delivering sgRNA to activate endogenous GSDME expression, combined with cisplatin release, induced pyroptosis and enhanced antitumor immunity, demonstrating a synergistic strategy leveraging gene editing and chemotherapy (Wang N. et al., 2023). Additionally, CRISPR-Cas9-mediated knockout of GSDME or caspase-3 in cancer cells modulated pyroptosis and apoptosis balance, influencing therapeutic outcomes (Xian et al., 2024; Tan et al., 2022). These studies underscore the versatility of gene editing and RNAi technologies in fine-tuning pyroptosis pathways for disease treatment.

Moreover, delivery systems facilitating gene editing and RNAi are critical for clinical translation. For example, albumin-based nanoparticles have been engineered to protect and deliver CRISPR-Cas9 components targeting PAR2, effectively mitigating cytokine storm by attenuating TLR4/NF-κB signaling and pyroptosis (Li J. et al., 2025). Similarly, α-synuclein-gold nanoparticle conjugates enable rapid and nondisruptive DNA transfection, offering a platform for delivering genes such as granzyme A to induce pyroptosis (Park et al., 2025). These innovative delivery modalities enhance the specificity and efficiency of gene editing and RNAi therapies targeting pyroptosis.

In addition to gene-editing and RNA-based approaches, enhancer elements have recently gained attention as important regulators of therapeutic gene expression. Natural tissue-specific enhancers and engineered synthetic enhancers have been incorporated into gene delivery constructs to achieve selective expression in particular cell types while minimizing off-target effects. Such strategies are particularly attractive for pyroptosis-related interventions, where excessive or systemic activation of inflammasome signaling may result in undesirable inflammatory toxicity (Najafi et al., 2024).

#### 2.4.5 Combined immunotherapy and pyroptosis induction

The integration of pyroptosis induction with ICB therapy represents a promising frontier in cancer treatment, aiming to overcome the limitations of current immunotherapies. Pyroptosis, a highly inflammatory form of programmed cell death mediated by GSDM proteins, enhances tumor immunogenicity by releasing TAAs, DAMPs, and proinflammatory cytokines such as IL-1β and IL-18. These factors collectively remodel the TME from an immunosuppressive “cold” state to an immunoreactive “hot” state, thereby potentiating the efficacy of ICIs like anti-PD-1/PD-L1 and CTLA-4 antibodies. Multiple studies have demonstrated that pyroptosis activation in tumor cells promotes DC maturation, increases infiltration of cytotoxic CD8^+^ T cells and NK cells, and reduces immunosuppressive cell populations within tumors, which synergistically enhance antitumor immunity and improve clinical outcomes (Wang W. et al., 2022; Bourne and Taabazuing, 2024; Zhang et al., 2024).

Recent advances in nanotechnology have enabled precise and controlled induction of pyroptosis within tumors, minimizing off-target effects and enhancing immunotherapeutic synergy. Nanomaterial-based delivery systems can co-deliver pyroptosis inducers and ICIs, or be engineered to respond to tumor-specific stimuli such as ROS or acidic pH, thereby achieving spatiotemporal control over pyroptosis activation. For instance, ROS/GSH dual-responsive nano-prodrugs combining chemotherapeutic agents and photosensitizers have been shown to induce GSDME-mediated pyroptosis upon photodynamic therapy, leading to enhanced DC maturation, increased cytotoxic T cell infiltration, and improved efficacy of ICB therapy (Xie et al., 2025; Ji et al., 2024; Xiao et al., 2021). Similarly, calcium ion nanomodulators and biodegradable upconversion nanoparticles have been developed to trigger mitochondrial calcium overload or ionic imbalances that activate pyroptosis and subsequent antitumor immune responses (Qi et al., 2025; Ding et al., 2021).

Clinical and preclinical data further support the prognostic and predictive value of pyroptosis-related signatures in various cancers, including glioma, bladder cancer, breast cancer, and lung squamous cell carcinoma. High pyroptosis scores correlate with increased infiltration of CD8^+^ T cells, elevated expression of immune checkpoints, and better responses to immunotherapy, suggesting that pyroptosis status can guide patient stratification and therapeutic decisions (Zeng et al., 2022; Wu et al., 2021b; Deng et al., 2022; Zhang Q. et al., 2022). Moreover, combinatory strategies that induce pyroptosis alongside ICB have demonstrated synergistic effects, converting immunologically “cold” tumors into “hot” ones and overcoming resistance to checkpoint blockade (Gao et al., 2022; Duan et al., 2025).

However, challenges remain in balancing the extent of pyroptosis induction to maximize antitumor immunity while minimizing systemic inflammation and tissue damage. Innovative approaches such as enzymatically switchable pyroptosis-inducing polymer conjugates and tunable nanomodulators offer precise control over pyroptosis levels, enhancing safety and therapeutic index (Chen et al., 2025; Ghosh et al., 2026). Additionally, understanding the interplay between pyroptosis and other forms of regulated cell death, as well as the epigenetic regulation of pyroptosis-related genes, may provide novel targets to further enhance immunotherapy outcomes (Zhang R. N. et al., 2023; Gao et al., 2022).

**FIGURE 4:**
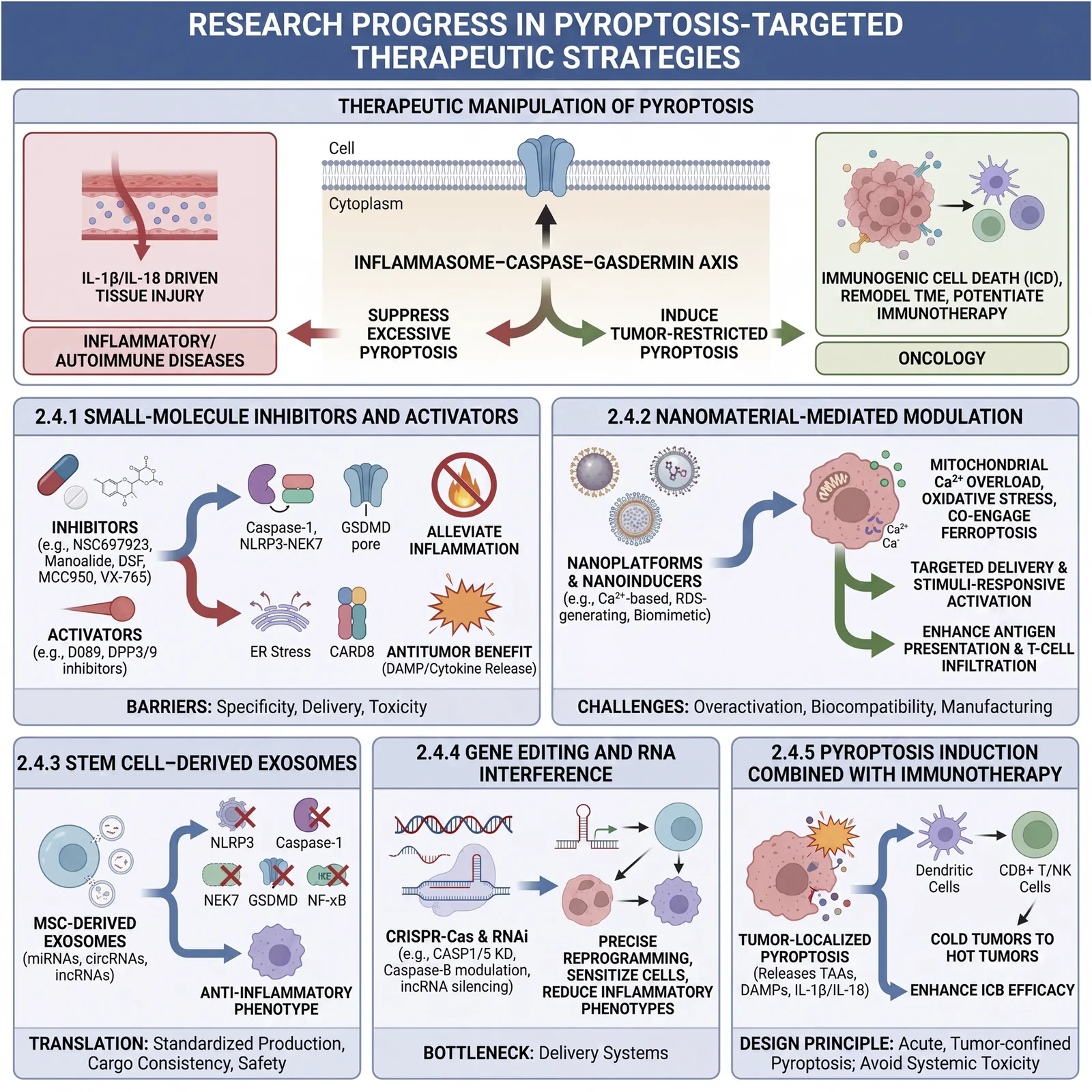
Multilevel regulation of pyroptosis.

In conclusion, the mechanistic crosstalk between pyroptosis and immune cells orchestrates a potent antitumor immune response. By promoting antigen release, inflammatory cytokine secretion, and immune cell activation, pyroptosis synergizes with immunotherapies to overcome tumor immune evasion (Figure 4). However, current pyroptosis-targeted therapeutic strategies differ substantially in their translational maturity. Small-molecule inhibitors of inflammasome- or cytokine-related pathways have the clearest clinical relevance, particularly because IL-1-targeted therapies and caspase-1 inhibitors provide human evidence that inflammasome-associated inflammation can be pharmacologically modulated. In contrast, nanomaterials, extracellular vesicles, and gene-regulation platforms offer greater tissue specificity and mechanistic flexibility but remain largely preclinical. Their clinical translation is still limited by delivery efficiency, biodistribution, manufacturing reproducibility, long-term safety, and the risk of excessive or off-target inflammatory activation.

Regulatory network controlling pyroptosis activation and execution. Pyroptosis is fine-tuned by upstream danger signals, intracellular signaling pathways, post-translational modifications, and epigenetic mechanisms, including ncRNAs. These regulatory layers determine the intensity, duration, and biological outcome of pyroptotic responses.

### 2.5 Application of pyroptosis in disease diagnosis and prognosis

#### 2.5.1 Screening and validation of pyroptosis-related biomarkers

Elevated expression levels of these GSDMs and cytokines have been clinically correlated with disease activity and prognosis. For instance, in Sjögren’s Syndrome, increased expression of GSDMD, along with pyroptosis-associated genes GZMA and GBP1, was observed in salivary gland tissues, and their combined diagnostic value was high (AUC = 0.8858), indicating their potential as biomarkers for inflammation-related pyroptosis (Zhang K. et al., 2023). Similarly, in bladder cancer, key pyroptosis-related genes including CASP8, NLRP3, and IL18 were found to be differentially expressed, and immune infiltration analysis revealed significant involvement of immune cells such as CD8^+^ T cells and macrophages, underscoring the clinical relevance of these biomarkers in tumor immunity and progression (Li P. et al., 2025). In RA, CASP8 emerged as a common biomarker linking ferroptosis and pyroptosis, with its expression validated in FLSs; furthermore, quercetin was identified as a potential therapeutic agent targeting CASP8, highlighting the translational potential of pyroptosis biomarkers (Zheng et al., 2023).

The integration of pyroptosis-related gene expression into scoring systems has enhanced prognostic assessments in oncology. In CRC, a pyroptosis-related gene signature including RSAD2 was developed, which independently predicted patient prognosis and correlated with TIME status, thereby offering a valuable prognostic biomarker and immunotherapy response indicator (Li Y. et al., 2024). Similarly, in gastric cancer, a pyroptosis risk score (PRS) constructed from four pyroptosis-related genes (BATF2, PTPRJ, RGS1, VCAN) demonstrated robust prognostic performance and was associated with immune cell infiltration patterns that predicted neoadjuvant immunotherapy efficacy (Wang J. B. et al., 2024). In TNBC, a pyroptosis scoring system (Ps-score) stratified patients into subtypes with distinct immunological features and therapeutic responses, validated by both bulk and single-cell RNA sequencing (scRNA-seq) data, emphasizing the clinical utility of pyroptosis-based prognostic models (Li et al., 2022d).

Moreover, in melanoma, a pyroptosis-based model constructed through LASSO regression effectively predicted clinical benefit from immunotherapy, with high pyroptosis scores correlating with an immune-inflamed TME and improved survival outcomes, thus serving as a reliable biomarker for treatment stratification (Wu G. et al., 2023). In lung adenocarcinoma, pyroptosis-related lncRNA signatures were also identified as prognostic biomarkers, further demonstrating the expanding scope of pyroptosis markers beyond protein-coding genes (Wang P. et al., 2024).

Emerging evidence suggests that pyroptosis-associated molecules may serve as valuable liquid biopsy biomarkers. Circulating gasdermin family proteins, inflammasome components, and inflammatory cytokines such as IL-1β and IL-18 reflect ongoing pyroptotic activity and have been associated with tumor progression, immune status, and therapeutic response. Elevated levels of these molecules have been reported in multiple malignancies and may provide complementary diagnostic information beyond conventional biomarkers (Salehnia et al., 2026).

#### 2.5.2 Development of pyroptosis activity detection techniques

The advancement of pyroptosis activity detection techniques has been pivotal in elucidating the molecular mechanisms and pathological roles of pyroptosis in various diseases, especially cancer and inflammatory disorders. Single-cell sequencing and multi-omics analysis technologies have emerged as powerful tools to dissect pyroptosis at cellular resolution and to integrate diverse molecular layers, providing comprehensive insights into pyroptosis regulation and its heterogeneity across cell types. For instance, scRNA-seq has been employed to identify pyroptosis-related gene expression signatures in specific immune cell populations within disease microenvironments. In necrotizing enterocolitis (NEC), scRNA-seq revealed increased macrophage abundance with a distinct cluster exhibiting elevated pyroptosis scores, highlighting the role of pyroptosis in disease progression and identifying TREM1 as a potential therapeutic target (Zhang P. et al., 2025). Similarly, in ischemic stroke, bioinformatics analysis combined with *in vivo* and *in vitro* experiments identified Annexin A3 (ANXA3) as a pyroptosis-related biomarker, with silencing of ANXA3 alleviating pyroptosis and microglial activation (Liu L. et al., 2023). These studies illustrate how single-cell transcriptomics, coupled with protein-level validation, can pinpoint cell-type-specific pyroptosis pathways and candidate regulators.

Multi-omics approaches, integrating transcriptomics, proteomics, epigenomics, and metabolomics, further enhance the understanding of pyroptosis mechanisms. These integrative analyses enable the identification of key genes like NLRP3, caspase-1, and GSDMs, and their regulatory networks, including ncRNAs, and epigenetic modifications, which govern pyroptotic responses. Moreover, molecular docking and dynamics simulations have been applied to predict and validate interactions between small molecules and pyroptosis-related proteins, facilitating drug discovery efforts (Lestari et al., 2025).

In the context of body fluid detection, several methods have been developed to monitor pyroptosis-related molecules non-invasively, which is crucial for clinical diagnosis and therapeutic monitoring. Enzyme-linked immunosorbent assays (ELISA) and Western blotting are routinely used to measure levels of pyroptosis-associated cytokines such as IL-1β and IL-18, as well as cleaved GSDM fragments in serum or plasma samples. For example, elevated serum IL-1β and IL-18 levels have been correlated with pyroptosis activation in sepsis, DN, and myocardial injury (Ye et al., 2025; Wang S. et al., 2024; Yao et al., 2025). Lactate dehydrogenase (LDH) release assays serve as indicators of cell membrane rupture during pyroptosis and have been used in both *in vitro* and *in vivo* studies to assess pyroptotic cell death (Duan et al., 2024; Huang et al., 2024). In addition, novel molecular probes and fluorescent reporters have been developed for live-cell imaging of pyroptosis, enabling real-time monitoring of caspase activation and GSDM pore formation (Ali and Wang, 2024; Wang X. et al., 2023). These probes, combined with near-infrared fluorescence imaging, have been successfully applied in cancer chemo-immunotherapy models to predict therapeutic efficacy by visualizing intratumoral pyroptosis (Wang X. et al., 2023).

Furthermore, bioinformatics analyses of circul ating extracellular vesicles and exosomes have identified pyroptosis-related miRNAs and lncRNAs as potential biomarkers detectable in body fluids, offering minimally invasive diagnostic options. For instance, exosomal miR-25-3p derived from platelet-rich plasma has been shown to alleviate chondrocyte pyroptosis in osteoarthritis, suggesting its potential as a therapeutic biomarker (Wang J. et al., 2025). Similarly, circulating histone levels correlated with endothelial pyroptosis and septic shock severity, providing a link between pyroptosis markers in blood and disease prognosis (Beltrán-García et al., 2022).

#### 2.5.3 Value of pyroptosis markers in monitoring therapeutic response

**FIGURE 5:**
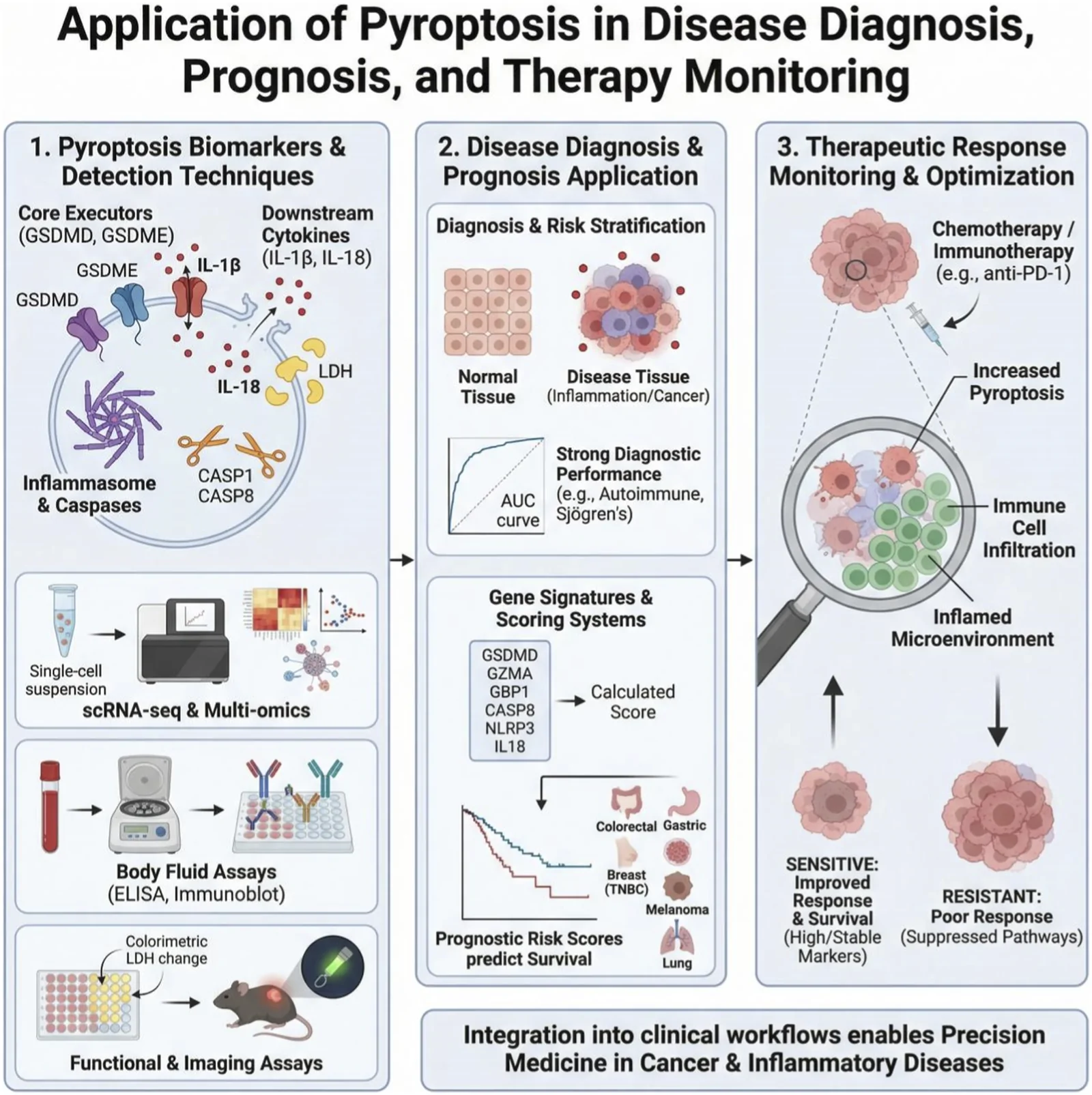
Therapeutic strategies targeting pyroptosis.

The quantification and dynamic monitoring of pyroptosis markers have emerged as promising tools for predicting and evaluating therapeutic responses in various diseases, particularly cancer and inflammatory conditions. Pyroptosis, an inflammatory form of programmed cell death mediated by gsdms and inflammasomes, plays a dual role in modulating treatment efficacy by directly inducing tumor cell death and shaping the immune microenvironment. Several studies highlight the predictive value of pyroptosis levels in assessing chemotherapy and immunotherapy outcomes. For instance, in cancer therapy, the expression of GSDM family members, especially GSDME and GSDMD, correlates with enhanced sensitivity to chemotherapeutic agents and ICIs. The deubiquitinating enzyme USP48 stabilizes GSDME, promoting pyroptosis and augmenting the efficacy of PD-1 blockade in tumor models, indicating that higher pyroptosis activity predicts better immunotherapeutic response (Ren Y. et al., 2023). Similarly, in head and neck squamous cell carcinoma, tumor-intrinsic SIRPA expression suppresses GSDME-mediated pyroptosis, reducing chemotherapy and immunotherapy effectiveness; thus, pyroptosis markers can stratify patients likely to benefit from these treatments (Song A. et al., 2025). Furthermore, pyroptosis-related gene signatures have been developed to classify patients into subgroups with distinct immune infiltration profiles and therapeutic responses. In glioma, a pyroptosis regulation score (PSscore) correlates with tumor mutational burden, immune cell infiltration, and response to anti-PD-1 therapy, underscoring its prognostic and predictive utility (Fan et al., 2022). In metastatic melanoma, pyroptosis-related gene scores associate with immune-enriched TMEs and improved immunotherapy outcomes (Chen et al., 2024b). These findings suggest that baseline pyroptosis levels can predict treatment efficacy, while longitudinal monitoring of pyroptosis dynamics can guide personalized therapy adjustments. For example, fluorescence probes detecting mitochondrial oxidative stress and pyroptosis activation provide real-time feedback on chemotherapy-induced tumor cell death, enabling precise therapeutic monitoring (Deng et al., 2025). In autoimmune and inflammatory diseases, urinary biomarkers linked to pyroptosis, such as vitronectin in juvenile SLE, offer non-invasive means to monitor disease activity and treatment response (Zhang et al., 2022c). Moreover, in sepsis and acute organ injuries, circulating pyroptosis markers like GSDMD and inflammasome components reflect disease progression and therapeutic efficacy, facilitating early intervention and prognosis assessment (Li et al., 2025e; Guo et al., 2026). Collectively, these studies demonstrate that pyroptosis markers serve as valuable indicators for predicting chemotherapy and immunotherapy outcomes and for dynamic monitoring to optimize individualized treatment strategies. Integration of pyroptosis biomarker profiling into clinical practice holds promise for enhancing precision medicine across oncology and inflammatory disorders (Figure 5).

Current and emerging therapeutic approaches targeting pyroptosis pathways. Strategies include inhibition of inflammasomes and GSDMs to suppress excessive inflammation, as well as deliberate induction of pyroptosis to enhance antitumor immunity. Nanomedicine, RNA-based therapies, and combination strategies further expand the therapeutic landscape.

### 2.6 Challenges and future prospects in pyrolysis research

#### 2.6.1 Safety and specificity issues in clinical translation

In cancer therapy, pyroptosis inducers like acevaltrate have demonstrated efficacy in overcoming drug resistance by promoting caspase-3/GSDME-dependent pyroptosis in myeloma cells, yet their pro-inflammatory nature raises concerns about systemic toxicity and cytokine release syndrome (Wang Y. et al., 2025). The risk of uncontrolled inflammation is also evident in sepsis, where pyroptosis contributes to multi-organ injury through excessive cytokine storm and tissue damage; thus, therapeutic modulation requires a delicate balance to avoid exacerbating systemic toxicity (Liu and Liang, 2025). Moreover, nanomaterials such as nanodiamonds have been reported to induce platelet pyroptosis and thrombocytopenia via NLRP3 inflammasome activation, highlighting the potential hematological side effects of pyroptosis-inducing agents (Hung et al., 2022). Natural compounds like luteolin and metformin have shown favorable safety profiles in modulating pyroptosis-related pathways, suggesting that careful selection and dosing of pyroptosis modulators can mitigate adverse effects (Sun et al., 2025; Zhang et al., 2021b). Nonetheless, the inflammatory amplification inherent to pyroptosis necessitates rigorous preclinical and clinical evaluation to minimize risks of excessive immune activation and off-target toxicity in patients. Achieving precise and stable delivery of pyroptosis modulators is paramount to enhancing therapeutic efficacy while minimizing systemic side effects. Conventional systemic administration often results in suboptimal drug concentrations at target tissues and off-target activation of pyroptosis, leading to safety concerns. To address this, advanced delivery platforms such as nanoparticles, liposomes, and engineered viral vectors have been developed to improve targeting specificity and pharmacokinetic stability. For instance, recombinant human heavy chain ferritin (HFn) nanocages loaded with 5-fluorouracil (5-FU@HFn) demonstrated selective uptake by chronic myeloid leukemia cells via CD71-mediated endocytosis, enhancing pyroptosis induction and reducing off-target toxicity (Yuan et al., 2025). Similarly, nanoliposomes responsive to pyroptotic cell signals have been engineered to release anti-pyroptotic drugs on-demand within inflamed tissues, exploiting GSDME interactions to achieve self-adaptive drug release and improved therapeutic outcomes in autoimmune inflammatory diseases (Xu et al., 2024). In cancer therapy, bioorthogonal pyroptosis nanoregulators facilitate *in situ* photosensitizer synthesis and autophagy inhibition specifically in tumor mitochondria, thereby enhancing pyroptosis efficacy while sparing normal tissues (Zhang W. et al., 2023). Moreover, erythrocyte membrane-camouflaged Pt (II) layered double hydroxide nanocages (RmPLH) prolong systemic circulation, evade immune clearance, and enable tumor-targeted sonodynamic therapy that synergizes with ICIs (Wu Y. et al., 2025). Viral delivery systems have also been optimized to induce pyroptosis selectively in tumor cells; for example, oncolytic adenoviruses armed with neutrophil elastase or PPE proteins promote macrophage polarization and effector T cell infiltration, enhancing antitumor immunity with controlled activation (Wang S. et al., 2025). Despite these advances, challenges remain in ensuring the stability of delivery vehicles under physiological conditions, avoiding premature drug release, and achieving precise spatiotemporal control over pyroptosis induction. Stability issues can lead to rapid drug degradation or unintended activation, compromising safety and efficacy. Therefore, ongoing research focuses on refining targeting ligands, stimuli-responsive materials, and combination strategies to enhance delivery precision and maintain therapeutic stability in clinical settings.

#### 2.6.2 Development and optimization of novel therapeutic strategies

Given pyroptosis’s complex involvement in tumor progression and immune modulation, combination therapies are designed to simultaneously target multiple nodes within the pyroptotic and associated signaling pathways to synergistically enhance antitumor immunity and overcome tumor resistance.

For instance, the integration of pyroptosis inducers with ICIs has shown promise in transforming immunologically “cold” tumors into “hot” ones by enhancing CD8^+^ T cell infiltration and activity (Ren Y. et al., 2023; Zhang Q. et al., 2022). Moreover, combinatorial regimens involving canonical pyroptosis activation (via caspase-1/GSDMD) alongside modulation of apoptosis or autophagy checkpoints have been proposed to circumvent tumor cell survival mechanisms that dampen pyroptotic efficacy (Zhang W. et al., 2023; Hu et al., 2023). The use of small-molecule inhibitors or activators targeting inflammasomes (e.g., NLRP3), caspases, and GSDM family members enables fine-tuning of pyroptotic responses, balancing proinflammatory effects to avoid excessive tissue damage while maximizing tumor cell death (Liu Q. et al., 2026; Shang et al., 2025).

Additionally, repurposing existing drugs with known pyroptosis-modulating properties, such as metformin, simvastatin, and curcumin, in combination with conventional chemotherapeutics, has been explored to potentiate pyroptosis-mediated tumor suppression while reducing systemic toxicity (Wu H. et al., 2022; Eldken et al., 2025). The identification of molecular regulators like USP48, which stabilizes GSDME to promote pyroptosis and enhance immunotherapy efficacy, further supports the rationale for multi-target combination approaches (Ren Y. et al., 2023). These strategies are underpinned by comprehensive molecular profiling and bioinformatics analyses to tailor therapies based on tumor-specific pyroptosis signatures and immune microenvironment characteristics, thereby advancing precision medicine paradigms (Ouyang et al., 2024; Liang et al., 2021).

Despite promising preclinical data, challenges remain in optimizing dosing regimens, minimizing off-target inflammation, and managing the dualistic nature of pyroptosis in cancer and inflammatory diseases. Future directions emphasize the integration of pyroptosis-targeting agents with immunomodulatory drugs, epigenetic modulators, and metabolic regulators to achieve sustained therapeutic responses with manageable safety profiles (Tuncer and Alcan, 2023; Hou et al., 2025). Overall, multi-target combination therapies hold significant potential to harness the full therapeutic benefit of pyroptosis modulation across diverse pathological contexts.

#### 2.6.3 Future research directions and technological innovations

The rapid expansion of pyroptosis research has revealed its pivotal role in diverse diseases, especially cancer and inflammatory disorders, positioning it as a promising therapeutic target. However, the complexity of pyroptosis pathways and the multifaceted roles of GSDM proteins necessitate advanced strategies to identify effective modulators. High-throughput screening (HTS) technologies offer powerful platforms to systematically evaluate large compound libraries for pyroptosis modulators, enabling the discovery of novel small molecules or biologics that can specifically induce or inhibit pyroptosis. Coupling HTS with artificial intelligence (AI) and machine learning algorithms further enhances drug discovery by enabling predictive modeling of compound-target interactions, optimizing lead compound selection, and accelerating the identification of candidates with favorable pharmacokinetic and safety profiles. Additionally, *in silico* methods integrating databases of pyroptosis-related genes, proteins, and pathways facilitate the identification of druggable targets and the rational design of inhibitors or activators (Zhang L. et al., 2025; Zhu et al., 2025). The integration of HTS and AI-assisted drug design holds great potential to overcome current challenges in pyroptosis-targeted therapy development, such as specificity, efficacy, and toxicity, ultimately accelerating the translation of pyroptosis modulators into clinical applications.

Emerging evidence highlights the intricate crosstalk between pyroptosis and immunometabolic pathways within the TME, which critically influences tumor progression, immune evasion, and therapeutic responses. Pyroptosis can modulate the release of proinflammatory cytokines and danger signals, reshaping the TME by affecting immune cell infiltration, angiogenesis, and stromal interactions (Wang H. et al., 2024; Liu W. et al., 2023). Immunometabolism, encompassing metabolic reprogramming of immune and tumor cells, governs the functional state of immune effectors and the inflammatory milieu. Studies suggest that metabolic intermediates and oxidative stress regulate pyroptosis activation, while pyroptosis in turn influences metabolic pathways, creating feedback loops that impact tumor immunity and inflammation (Wang J. et al., 2024; Cao et al., 2023). Comprehensive mechanistic investigations are needed to elucidate how pyroptosis intersects with immunometabolic networks and TME components, including cancer-associated fibroblasts, MDSCs, and Tregs.

**FIGURE 6:**
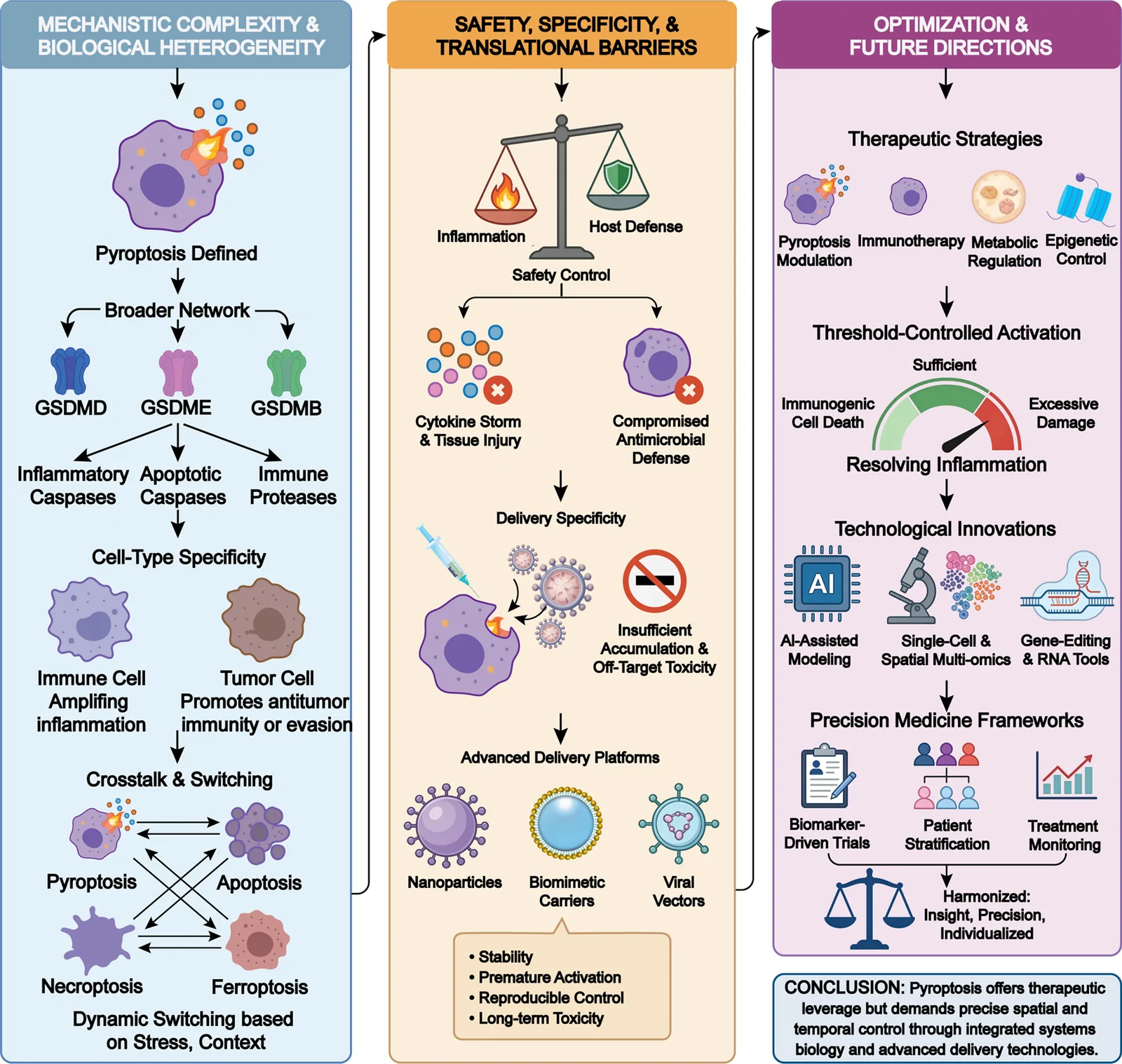
Translational challenges and future perspectives.

Translating pyroptosis research into clinical practice requires well-designed clinical trials that account for the heterogeneity of diseases and patient-specific factors. Given the dual roles of pyroptosis in disease pathogenesis—both protective and detrimental depending on context—precision medicine approaches are essential. Biomarker-driven stratification based on pyroptosis-related gene expression profiles, inflammasome activity, and GSDM expression can identify patient subgroups likely to benefit from pyroptosis-targeted therapies (Zhou et al., 2021; Chen et al., 2023). Incorporation of pyroptosis scoring systems and molecular signatures into trial design can optimize inclusion criteria and therapeutic monitoring. Moreover, integrating multi-omics data and AI-driven predictive models can guide individualized treatment regimens that combine pyroptosis modulators with immunotherapies, chemotherapy, or targeted agents. The development of novel delivery systems, such as nanomedicine platforms and MSC-Exos, offers opportunities for targeted and controlled modulation of pyroptosis in specific tissues, enhancing efficacy and minimizing systemic toxicity (Yu et al., 2025; Yang et al., 2025). Future clinical trials should also consider the timing and dosing of pyroptosis-inducing agents to balance antitumor immunity and inflammation-related adverse effects. Collectively, these strategies will facilitate the construction of personalized therapeutic regimens, maximizing clinical benefits while mitigating risks associated with pyroptosis modulation (Figure 6).

Challenges and future directions for pyroptosis-based therapies. Key considerations include disease-specific heterogeneity, safety concerns related to excessive inflammation, precision targeting, and patient stratification. Addressing these challenges is essential for translating pyroptosis modulation into clinical practice.

## 3 Conclusion

Pyroptosis has emerged as a pivotal form of inflammatory programmed cell death that influences a broad spectrum of diseases, including cancer, cardiovascular disorders, metabolic diseases, and autoimmune conditions. Growing evidence indicates that pyroptosis functions as a double-edged sword: while controlled activation can promote host defense, tissue repair, and antitumor immunity, excessive or chronic pyroptosis may drive pathological inflammation and disease progression. The ultimate biological outcome is highly context dependent and influenced by factors such as disease type, disease stage, genetic background, and immune microenvironment composition.

Recent advances in pyroptosis research have identified numerous therapeutic opportunities, including small-molecule inhibitors, extracellular vesicle-based therapies, nanomedicine platforms, gene regulation technologies, and emerging precision drug-delivery systems. In parallel, pyroptosis-related biomarkers, scoring systems, and liquid biopsy approaches are showing increasing promise for disease diagnosis, prognostic stratification, treatment monitoring, and prediction of therapeutic responses.

Despite these advances, several challenges remain. Most current evidence is derived from preclinical studies, while prospective clinical validation remains limited. In addition, substantial heterogeneity exists in pyroptosis biomarkers and assessment methods, and the complex crosstalk between pyroptosis and other cell death pathways continues to complicate mechanistic interpretation and therapeutic targeting.

Future studies should focus on defining the context-specific determinants of pyroptosis, establishing standardized biomarkers, and translating emerging therapeutic strategies into clinically validated interventions. Such efforts will be essential for harnessing the full potential of pyroptosis in precision medicine and next-generation therapeutic development.

## References

[B1] Al Mamun A. Mimi A. A. Aziz M. A. Zaeem M. Ahmed T. Munir F. (2021a). Role of pyroptosis in cancer and its therapeutic regulation. Eur. J. Pharmacol. 910, 174444. 10.1016/j.ejphar.2021.174444 34453928

[B2] Al Mamun A. Ara Mimi A. Wu Y. Zaeem M. Abdul Aziz M. Aktar Suchi S. (2021b). Pyroptosis in diabetic nephropathy. Clin. Chim. Acta 523, 131–143. 10.1016/j.cca.2021.09.003 34529985

[B3] Ali W. Wang F. (2024). Molecular probes for monitoring pyroptosis: design, imaging and theranostic application. Apoptosis 29, 1038–1050. 10.1007/s10495-024-01980-3 38772991

[B4] Anderson M. J. den Hartigh A. B. Loomis W. P. Fink S. L. (2024). Broad-spectrum inflammasome inhibition by thiomuscimol. Cell. Death Discov. 10, 470. 10.1038/s41420-024-02238-2 39550359 PMC11569204

[B5] Bai B. Yang Y. Ji S. Wang S. Peng X. Tian C. (2021). MicroRNA-302c-3p inhibits endothelial cell pyroptosis *via* directly targeting NOD-LRR- and pyrin domain-containing protein 3 in atherosclerosis. J. Cell. Mol. Med. 25, 4373–4386. 10.1111/jcmm.16500 33783966 PMC8093969

[B6] Bai Z. Hu H. Hu F. Ji J. Ji Z. (2023). Bone marrow mesenchymal stem cellsderived exosomes stabilize atherosclerosis through inhibiting pyroptosis. BMC Cardiovasc Disord. 23, 441. 10.1186/s12872-023-03453-y 37679676 PMC10486039

[B7] Bai Y. Pan Y. Liu X. (2025). Mechanistic insights into gasdermin-mediated pyroptosis. Nat. Rev. Mol. Cell. Biol. 26, 501–521. 10.1038/s41580-025-00837-0 40128620

[B8] Bandharam N. Lockey R. F. Kolliputi N. (2023). Pyroptosis inhibition in disease treatment: opportunities and challenges. Cell. Biochem. Biophys. 81, 615–619. 10.1007/s12013-023-01181-w 37782424

[B9] Bao X. Sun M. Meng L. Zhang H. Yi X. Zhang P. (2024). Applications of pyroptosis activators in tumor immunotherapy. Mater Today Bio 28, 101191. 10.1016/j.mtbio.2024.101191 PMC11363858 39221221

[B10] Beltrán-García J. Osca-Verdegal R. Pérez-Cremades D. Novella S. Hermenegildo C. Pallardó F. V. (2022). Extracellular histones activate endothelial NLRP3 inflammasome and are associated with a severe sepsis phenotype. J. Inflamm. Res. 15, 4217–4238. 10.2147/jir.s363693 35915852 PMC9338392

[B11] Berkel C. Cacan E. (2023). Pollutant-induced pyroptosis in humans and other animals. Life Sciences 316, 121386. 10.1016/j.lfs.2023.121386 36657639

[B12] Bertheloot D. Latz E. Franklin B. S. (2021). Necroptosis, pyroptosis and apoptosis: an intricate game of cell death. Cell. Mol. Immunol. 18, 1106–1121. 10.1038/s41423-020-00630-3 33785842 PMC8008022

[B13] Bin T. Lin C. Liu F. J. Wang Y. Xu X. J. Lin D. J. (2022). Establishment of a risk model correlated with metabolism based on RNA-binding proteins associated with cell pyroptosis in acute myeloid leukemia. Front. Oncol. 12, 1059978. 10.3389/fonc.2022.1059978 36465357 PMC9713014

[B14] Booty L. M. Bryant C. E. (2022). Gasdermin D and beyond - gasdermin-mediated pyroptosis in bacterial infections. J. Mol. Biol. 434, 167409. 10.1016/j.jmb.2021.167409 34929200

[B15] Bourne C. M. Taabazuing C. Y. (2024). Harnessing pyroptosis for cancer immunotherapy. Cells 13, 346. 10.3390/cells13040346 38391959 PMC10886719

[B16] Burdette B. E. Esparza A. N. Zhu H. Wang S. (2021). Gasdermin D in pyroptosis. Acta Pharm. Sin. B 11, 2768–2782. 10.1016/j.apsb.2021.02.006 34589396 PMC8463274

[B17] Cai Z. Yuan S. Luan X. Feng J. Deng L. Zuo Y. (2022). Pyroptosis-related inflammasome pathway: a new therapeutic target for diabetic cardiomyopathy. Front. Pharmacol. 13, 842313. 10.3389/fphar.2022.842313 35355717 PMC8959892

[B18] Cai Y. Li K. Lin J. Liang X. Xu W. Zhan Z. (2022). Lighting a fire: gasdermin-mediated pyroptosis remodels the glioma microenvironment and promotes immune checkpoint blockade response. Front. Immunol. 13, 910490. 10.3389/fimmu.2022.910490 35784306 PMC9249059

[B19] Cai D. Li C. Zhang Y. He S. Guo Y. Liao W. (2024). CircHipk3 serves a dual role in macrophage pyroptosis by promoting NLRP3 transcription and inhibition of autophagy to induce abdominal aortic aneurysm formation. Clin. Transl. Med. 14, e70102. 10.1002/ctm2.70102 39601144 PMC11599875

[B20] Cao D. Y. Zhang Z. H. Li R. Z. Shi X. K. Xi R. Y. Zhang G. L. (2022). A small molecule inhibitor of caspase-1 inhibits NLRP3 inflammasome activation and pyroptosis to alleviate gouty inflammation. Immunol. Lett. 244, 28–39. 10.1016/j.imlet.2022.03.003 35288207

[B21] Cao Q. Zhang J. B. Sun D. Y. Fu J. T. Wu W. B. Chen X. F. (2023). Pyroptosis, metabolism, and oxidation in tumorigenesis: mechanisms and therapeutic implications. Antioxid. Redox Signal 39, 512–530. 10.1089/ars.2023.0257 36851903

[B22] Chai R. Li Y. Shui L. Ni L. Zhang A. (2023). The role of pyroptosis in inflammatory diseases. Front. Cell. Dev. Biol. 11, 1173235. 10.3389/fcell.2023.1173235 37250902 PMC10213465

[B23] Chai Q. Lei Z. Liu C. H. (2023). Pyroptosis modulation by bacterial effector proteins. Semin. Immunol. 69, 101804. 10.1016/j.smim.2023.101804 37406548

[B24] Chen X. Chen H. Yao H. Zhao K. Zhang Y. He D. (2021). Turning up the heat on non-immunoreactive tumors: pyroptosis influences the tumor immune microenvironment in bladder cancer. Oncogene 40, 6381–6393. 10.1038/s41388-021-02024-9 34588621

[B25] Chen X. Xu Y. Wang M. Ren C. (2023). Elucidating the role of pyroptosis in lower-grade glioma: development of a novel scoring system to enhance personalized therapeutic approaches. J. Mol. Neurosci. 73, 649–663. 10.1007/s12031-023-02147-6 37566191

[B26] Chen Y. Zeng D. Wei G. Liao Z. Liang R. Huang X. (2024). Pyroptosis in osteoarthritis: molecular mechanisms and therapeutic implications. J. Inflamm. Res. 17, 791–803. 10.2147/jir.s445573 38348279 PMC10860821

[B27] Chen C. Wang J. Zhang S. Zhu X. Hu J. Liu C. (2024). Epigenetic regulation of diverse regulated cell death modalities in cardiovascular disease: insights into necroptosis, pyroptosis, ferroptosis, and cuproptosis. Redox Biol. 76, 103321. 10.1016/j.redox.2024.103321 39186883 PMC11388786

[B28] Chen T. Liang K. Wang J. Li J. Xue X. Hao Y. (2024). An aged tree with a new bloom: a simple spatiotemporal programming strategy enables carbon dot photosensitizers to regulate cell pyroptosis for enhanced tumor photodynamic-immunotherapy. Nano Lett. 24, 14709–14719. 10.1021/acs.nanolett.4c03913 39504147

[B29] Chen W. Cheng J. Cai Y. Wang P. Jin J. (2024a). The pyroptosis-related signature predicts prognosis and influences the tumor immune microenvironment in dedifferentiated liposarcoma. Open Med. (Wars) 19, 20230886. 10.1515/med-2023-0886 38221934 PMC10787309

[B30] Chen M. Huang M. Chen X. Lin X. Chen X. (2024). Multiomics blueprint of PANoptosis in deciphering immune characteristics and prognosis stratification of glioma patients. Journal Gene Medicine 26, e3621. 10.1002/jgm.3621 37997255

[B31] Chen Q. Wang L. Wei X. Chen M. Zhang X. Mo R. (2024). Puerarin alleviates diabetic nephropathy by inhibiting Caspase-1-mediated pyroptosis. J. Pharm. Pharmacol. 76, 213–223. 10.1093/jpp/rgad113 38215026

[B32] Chen W. He Y. Zhou G. Chen X. Ye Y. Zhang G. (2024b). Multiomics characterization of pyroptosis in the tumor microenvironment and therapeutic relevance in metastatic melanoma. BMC Med. 22, 24. 10.1186/s12916-023-03175-0 38229080 PMC10792919

[B33] Chen Z. Yang Z. Rao Z. Luo Y. Liu W. Qiao C. (2025). A pyroptosis proportion tunable nano-modulator for cancer immunotherapy. Theranostics 15, 8320–8336. 10.7150/thno.112209 40860132 PMC12374590

[B34] Chen S. Zhu Y. Chen T. Xu Y. Fan Q. (2026). CSB6B attenuates renal inflammation and fibrosis by inhibiting the activation of NLRP3 inflammasome through the NLRP3/Caspase-1/GSDMD/IL-1β signaling pathway. Pathol. Res. Pract. 277, 156286. 10.1016/j.prp.2025.156286 41240443

[B35] Cheng H. Zhang Q. Zhong W. Li H. Wang L. Wang S. (2025). Pulsed electromagnetic fields inhibit atherosclerosis by regulating pyroptosis through membrane tension-mediated mechanosensitive channels. Signal Transduct. Target Ther. 10, 388. 10.1038/s41392-025-02479-2 41309549 PMC12660931

[B36] Deng X. Wang Z. Luo Y. Li Z. Chen L. (2022). Prediction of lung squamous cell carcinoma immune microenvironment and immunotherapy efficiency with pyroptosis-derived genes. Med. Baltim. 101, e30304. 10.1097/md.0000000000030304 36123889 PMC9478317

[B37] Deng M. Wang P. Zhai Z. Liu Y. Cheng D. He L. (2025). A triple-responsive and Dual-NIR emissive fluorescence probe for precise cancer imaging and therapy by activating pyroptosis pathway. Anal. Chem. 97, 2998–3008. 10.1021/acs.analchem.4c06015 39888040

[B38] Dessouki F. B. A. Kukreja R. C. Singla D. K. (2020). Stem cell-derived exosomes ameliorate doxorubicin-induced muscle toxicity through counteracting pyroptosis. Pharm. (Basel) 13, 450. 10.3390/ph13120450 PMC7764639 33316945

[B39] Ding B. Sheng J. Zheng P. Li C. Li D. Cheng Z. (2021). Biodegradable upconversion nanoparticles induce pyroptosis for cancer immunotherapy. Nano Lett. 21, 8281–8289. 10.1021/acs.nanolett.1c02790 34591494

[B40] Duan Y. Zhang L. Angosto-Bazarra D. Pelegrín P. Núñez G. He Y. (2020). RACK1 mediates NLRP3 inflammasome activation by promoting NLRP3 active conformation and inflammasome assembly. Cell. Rep. 33, 108405. 10.1016/j.celrep.2020.108405 33207200 PMC7709964

[B41] Duan L. J. Zhuang Q. Chen L. Zeng Y. Liu X. Y. Wang L. L. (2024). Effect and mechanism of free total rhubarb anthraquinones on pyroptosis of J774A.1 macrophages. Zhongguo Zhong Yao Za Zhi 49, 2210–2221. 10.19540/j.cnki.cjcmm.20231212.705 38812236

[B42] Duan W. Hosea R. Wang L. Ruan C. Zhao F. Liu J. (2025). Chromosome missegregation triggers tumor cell pyroptosis and enhances anti-tumor immunotherapy in colorectal cancer. Adv. Sci. (Weinh) 12, e2409769. 10.1002/advs.202409769 39903759 PMC11948012

[B43] Dubyak G. R. Miller B. A. Pearlman E. (2023). Pyroptosis in neutrophils: multimodal integration of inflammasome and regulated cell death signaling pathways. Immunol. Rev. 314, 229–249. 10.1111/imr.13186 36656082 PMC10407921

[B44] Eldken Z. H. Youssef O. M. Khalil R. M. Kassab A. A. M. Ramadan A. Sakr N. H. (2025). Curcumin's dual effect on pyroptosis and autophagy in metabolic-associated fatty liver disease: a potential therapeutic strategy. Sci. Rep. 15, 34309. 10.1038/s41598-025-11135-2 41038833 PMC12491484

[B45] Fan T. Wan Y. Niu D. Wang B. Zhang B. Zhang Z. (2022). Comprehensive analysis of pyroptosis regulation patterns and their influence on tumor immune microenvironment and patient prognosis in glioma. Discov. Oncol. 13, 13. 10.1007/s12672-022-00474-5 35274175 PMC8913830

[B46] Fan H. Xu P. Zou B. Wang H. Li C. Huang J. (2025). Isoquercitrin inhibits lung cancer cell growth through triggering pyroptosis and ferroptosis. Nutr. Cancer 77, 299–310. 10.1080/01635581.2024.2416246 39427296

[B47] Fang Y. Tang Y. Huang B. (2023). Pyroptosis: a road to next-generation cancer immunotherapy. Seminars Immunology 68, 101782. 10.1016/j.smim.2023.101782 37302166

[B48] Feng Y. Li M. Yangzhong X. Zhang X. Zu A. Hou Y. (2022). Pyroptosis in inflammation-related respiratory disease. J. Physiol. Biochem. 78, 721–737. 10.1007/s13105-022-00909-1 35819638 PMC9684248

[B49] Fingelkurts A. A. Fingelkurts A. A. (2019). Placing pure experience of eastern tradition into the neurophysiology of Western tradition. Cogn. Neurodynamics 13, 121–123. 10.1007/s11571-018-9506-0 30728875 PMC6339859

[B50] Fu X. Alkhamessi R. N. Al-Aouadi R. F. A. Kadham M. J. Khasanova S. Shree M. (2026). Inflammation-related lncRNAs in the regulation of kidney injuries; special emphasis on novel lncRNA-based delivery platforms. Naunyn Schmiedeb. Arch. Pharmacol. 399, 1793–1813. 10.1007/s00210-025-04516-x 40888877

[B51] Gaikwad S. M. Phyo Z. Arteaga A. Q. Gorjifard S. Calabrese D. R. Connors D. (2020). A small molecule stabilizer of the MYC G4-Quadruplex induces endoplasmic reticulum stress, senescence and pyroptosis in multiple myeloma. Cancers (Basel) 12, 2952. 10.3390/cancers12102952 33066043 PMC7650714

[B52] Gao W. Wang X. Zhou Y. Wang X. Yu Y. (2022). Autophagy, ferroptosis, pyroptosis, and necroptosis in tumor immunotherapy. Signal Transduct. Target Ther. 7, 196. 10.1038/s41392-022-01046-3 35725836 PMC9208265

[B53] Gao J. Wang D. Yang Q. Tang M. Du J. He L. (2023). The signature of pyroptosis-related gene prognostic and immune microenvironment in adrenocortical carcinoma. Front. Molecular Biosciences 10, 1131402. 10.3389/fmolb.2023.1131402 PMC9998516 36911522

[B54] Ge J. Zhang Z. Zhao S. Chen Y. Min X. Cai Y. (2024). Nanomedicine-induced cell pyroptosis to enhance antitumor immunotherapy. J. Mater Chem. B 12, 3857–3880. 10.1039/d3tb03017b 38563315

[B55] Ghaneialvar H. Jahani S. Hashemi E. Khalilzad M. A. Falahi S. Rashidi M. A. (2025). Combining anti-checkpoint immunotherapies and cancer vaccines as a novel strategy in oncological therapy: a review. Hum. Immunology 86, 111209. 10.1016/j.humimm.2024.111209 39662393

[B56] Ghosh T. Jeon J. Duong V. H. Ghahari F. Song S. H. Kim J. (2026). Enzymatically switchable pyroptosis-inducing polymer conjugate to coordinate host immune responses in cancer immunotherapy. Adv. Mater 38, e10103. 10.1002/adma.202510103 41321150

[B57] Gong X. Gu W. Fu S. Zou G. Jiang Z. (2024). Zinc homeostasis regulates caspase activity and inflammasome activation. PLoS Pathog. 20, e1012805. 10.1371/journal.ppat.1012805 39689159 PMC11687882

[B58] Gong W. Fu H. Yang K. Zheng T. Guo K. Zhao W. (2024). 4-Octyl itaconate blocks GSDMB-mediated pyroptosis and restricts inflammation by inactivating granzyme A. Cell. Prolif. 57, e13711. 10.1111/cpr.13711 38982510 PMC11628737

[B59] Guo Y. Wang Y. Wang B. Peng S. Li N. Zhang D. (2026). Ultrasound-responsive renal-targeted nanoparticles deliver TAK-242 to inhibit NF-κB/NLRP3 signaling and attenuate sepsis-associated acute kidney injury. Biomaterials 329, 123922. 10.1016/j.biomaterials.2025.123922 41481962

[B60] Han J. Zuo Z. Shi X. Zhang Y. Peng Z. Xing Y. (2023). Hirudin ameliorates diabetic nephropathy by inhibiting Gsdmd-mediated pyroptosis. Cell. Biol. Toxicol. 39, 573–589. 10.1007/s10565-021-09622-z 34212273

[B61] He Y. Ding D. Guo J. Sun H. Yang J. (2025). Activation and regulation of NAIP/NLRC4 and NLRP3 inflammasomes during salmonella infection: mechanisms and therapeutic implications. Mol. Biol. Rep. 53, 21. 10.1007/s11033-025-11166-y 41160185

[B62] Hendawy N. El-Sayed S. M. AbdelWahed D. M. Salaheldin S. H. Abuelezz S. A. (2025). Empagliflozin attenuates pyroptosis by regulating thioredoxin-NLRP3 inflammasome axis in a high fat cholesterol diet/streptozotocin model of atherosclerosis. J. Pharm. Pharmacol. 77, 1414–1425. 10.1093/jpp/rgaf051 40755206

[B63] Hou J. Li T. Hsu J. M. Zhang X. Hung M. C. (2023). Gasdermins and cancers. Semin. Immunol. 70, 101833. 10.1016/j.smim.2023.101833 37647772

[B64] Hou Y. Li W. Yang J. Yu H. Wang C. Li Y. (2025). Is pyroptosis a brake or an accelerator in the fate of the tumor? Cell. Death Dis. 16, 568. 10.1038/s41419-025-07866-9 40721578 PMC12304206

[B65] Hsu S. K. Li C. Y. Lin I. L. Syue W. J. Chen Y. F. Cheng K. C. (2021). Inflammation-related pyroptosis, a novel programmed cell death pathway, and its crosstalk with immune therapy in cancer treatment. Theranostics 11, 8813–8835. 10.7150/thno.62521 34522213 PMC8419056

[B66] Hu Y. Liu Y. Zong L. Zhang W. Liu R. Xing Q. (2023). The multifaceted roles of GSDME-mediated pyroptosis in cancer: therapeutic strategies and persisting obstacles. Cell. Death Dis. 14, 836. 10.1038/s41419-023-06382-y 38104141 PMC10725489

[B67] Huang Y. Li X. Luo G. Wang J. Li R. Zhou C. (2022a). Pyroptosis as a candidate therapeutic target for alzheimer's disease. Front. Aging Neurosci. 14, 996646. 10.3389/fnagi.2022.996646 36185484 PMC9520296

[B68] Huang Y. Wang J. W. Huang J. Tang L. Xu Y. H. Sun H. (2022b). Pyroptosis, a target for cancer treatment? Apoptosis 27, 1–13. 10.1007/s10495-021-01703-y 35064425

[B69] Huang X. Wang Y. Yang W. Dong J. Li L. (2022). Regulation of dietary polyphenols on cancer cell pyroptosis and the tumor immune microenvironment. Front. Nutr. 9, 974896. 10.3389/fnut.2022.974896 36091247 PMC9453822

[B70] Huang T. Ding J. Lin L. Han L. Yu L. Li M. (2023). Bioinformatic identification of the pyroptosis-related transcription Factor-MicroRNA-Target gene regulatory network in angiotensin II-Induced cardiac remodeling and validation of key components. Front. Biosci. Landmark Ed. 28, 293. 10.31083/j.fbl2811293 38062833

[B71] Huang H. Qiao Y. Chu L. Ye C. Lin L. Liao H. (2024). Up-regulation of HSP90α in HDM-induced asthma causes pyroptosis of airway epithelial cells by activating the cGAS-STING-ER stress pathway. Int. Immunopharmacol. 131, 111917. 10.1016/j.intimp.2024.111917 38527402

[B72] Huang D. Xu D. You C. Li B. Li L. Yang Q. (2025). Caspase-8-driven NLRP3 inflammasome activation exacerbates bronchopulmonary dysplasia by increasing the apoptosis and pyroptosis crosstalk of alveolar epithelial cells. Int. Immunopharmacol. 161, 115025. 10.1016/j.intimp.2025.115025 40483833

[B73] Humphries F. Shmuel-Galia L. Ketelut-Carneiro N. Li S. Wang B. Nemmara V. V. (2020). Succination inactivates gasdermin D and blocks pyroptosis. Science 369, 1633–1637. 10.1126/science.abb9818 32820063 PMC8744141

[B74] Hung S. C. Ke L. C. Lien T. S. Huang H. S. Sun D. S. Cheng C. L. (2022). Nanodiamond-induced thrombocytopenia in mice involve P-Selectin-Dependent Nlrp3 inflammasome-mediated platelet aggregation, pyroptosis and apoptosis. Front. Immunol. 13, 806686. 10.3389/fimmu.2022.806686 35444640 PMC9013758

[B75] Huston H. C. Anderson M. J. Fink S. L. (2023). Pyroptosis and the cellular consequences of gasdermin pores. Semin. Immunol. 69, 101803. 10.1016/j.smim.2023.101803 37437353 PMC10530493

[B76] Inkol J. M. Westerveld M. J. Verburg S. G. Walsh S. R. Morrison J. Mossman K. L. (2024). Pyroptosis activates conventional type I dendritic cells to mediate the priming of highly functional anticancer T cells. J. Immunother. Cancer 12, e006781. 10.1136/jitc-2023-006781 38580330 PMC11002387

[B77] Ji X. Huang X. Li C. Guan N. Pan T. Dong J. (2023). Effect of tumor-associated macrophages on the pyroptosis of breast cancer tumor cells. Cell. Commun. Signal 21, 197. 10.1186/s12964-023-01208-y 37542283 PMC10401873

[B78] Ji F. Shi C. Shu Z. Li Z. (2024). Nanomaterials enhance pyroptosis-based tumor immunotherapy. Int. J. Nanomedicine 19, 5545–5579. 10.2147/ijn.s457309 38882539 PMC11178094

[B79] Jiang S. Zhou Z. Sun Y. Zhang T. Sun L. (2020). Coral gasdermin triggers pyroptosis. Sci. Immunol. 5, eabd2591. 10.1126/sciimmunol.abd2591 33277371

[B80] Jiang Y. Song S. Liu J. Zhang L. Guo X. Lu J. (2023). Epigenetic regulation of programmed cell death in hypoxia-induced pulmonary arterial hypertension. Front. Immunol. 14, 1206452. 10.3389/fimmu.2023.1206452 37753070 PMC10518698

[B81] Johnson D. E. Cui Z. (2025). Triggering pyroptosis in cancer. Biomolecules 15, 348. 10.3390/biom15030348 40149884 PMC11940180

[B82] Ju X. Yang Z. Zhang H. Wang Q. (2021). Role of pyroptosis in cancer cells and clinical applications. Biochimie 185, 78–86. 10.1016/j.biochi.2021.03.007 33746064

[B83] Kaneko N. Kurata M. Yamamoto T. Sakamoto A. Takada Y. Kosako H. (2024). CANE, a component of the NLRP3 inflammasome, promotes inflammasome activation. J. Immunol. 213, 86–95. 10.4049/jimmunol.2300175 38787200

[B84] Khalilzad M. A. Mohammadi J. Najafi S. Amirsaadat S. Zare S. Khalilzad M. (2025a). Harnessing the anti-inflammatory effects of perinatal tissue derived therapies for the treatment of inflammatory skin diseases: a comprehensive review. Stem Cell Reviews Reports 21, 351–371. 10.1007/s12015-024-10822-3 39531196

[B85] Khalilzad M. A. Mohammadi J. Amirsaadat S. Najafi S. Zare S. Nilforoushzadeh M. A. (2025b). Elevating dermatology beyond aesthetics: perinatal-derived advancements for rejuvenation, alopecia strategies, scar therapies, and progressive wound healing. Stem Cell Reviews Reports 21, 709–729. 10.1007/s12015-024-10835-y 39804520

[B86] Khojali W. M. A. Khalifa N. E. Alshammari F. Afsar S. Aboshouk N. A. M. Khalifa A. A. S. (2024). Pyroptosis-related non-coding RNAs emerging players in atherosclerosis pathology. Pathol. Res. Pract. 255, 155219. 10.1016/j.prp.2024.155219 38401375

[B87] Kuo C. W. Su P. L. Huang T. H. Lin C. C. Chen C. W. Tsai J. S. (2023). Cigarette smoke increases susceptibility of alveolar macrophages to SARS-CoV-2 infection through inducing reactive oxygen species-upregulated angiotensin-converting enzyme 2 expression. Sci. Reports 13, 7894. 10.1038/s41598-023-34785-6 PMC10185955 37193781

[B88] Lestari B. Utomo R. Y. Rahman F. A. Putri D. D. P. Zulfin U. M. Suenaga Y. (2025). Discovery of pyroptosis-inducing natural products in neuroblastomas: computational studies with experimental validation. BMC Complement. Med. Ther. 25, 279. 10.1186/s12906-025-05004-8 40684133 PMC12276684

[B89] Li Y. Guo B. (2025). GSDMD-mediated pyroptosis: molecular mechanisms, diseases and therapeutic targets. Mol. Biomed. 6, 11. 10.1186/s43556-025-00249-8 39994107 PMC11850691

[B90] Li Y. Jiang Q. (2023). Uncoupled pyroptosis and IL-1β secretion downstream of inflammasome signaling. Front. Immunol. 14, 1128358. 10.3389/fimmu.2023.1128358 37090724 PMC10117957

[B91] Li Y. Pu D. Huang J. Zhang Y. Yin H. (2022). Protein phosphatase 1 regulates phosphorylation of gasdermin D and pyroptosis. Chem. Commun. (Camb) 58, 11965–11968. 10.1039/d2cc03590a 36205355

[B92] Li J. Liu J. Yu Y. Liu Y. Guan X. (2022). NF-κB/ABCA1 pathway aggravates ox-LDL-induced cell pyroptosis by activation of NLRP3 inflammasomes in THP-1-derived macrophages. Mol. Biol. Rep. 49, 6161–6171. 10.1007/s11033-022-07408-y 35579737

[B93] Li C. Lin H. He H. Ma M. Jiang W. Zhou R. (2022c). Inhibition of the NLRP3 inflammasome activation by manoalide ameliorates experimental autoimmune encephalomyelitis pathogenesis. Front. Cell. Dev. Biol. 10, 822236. 10.3389/fcell.2022.822236 35252186 PMC8888861

[B94] Li C. Pan J. Jiang Y. Wu Y. Jin Z. Chen X. (2022d). Characterization of pyroptosis-related subtypes *via* RNA-seq and ScRNA-Seq to predict chemo-immunotherapy response in triple-negative breast cancer. Front. Genet. 13, 788670. 10.3389/fgene.2022.788670 35386285 PMC8978671

[B95] Li M. Jiang P. Yang Y. Xiong L. Wei S. Wang J. (2023). The role of pyroptosis and gasdermin family in tumor progression and immune microenvironment. Exp. Hematol. Oncol. 12, 103. 10.1186/s40164-023-00464-5 38066523 PMC10704735

[B96] Li Z. Yang Z. Zhu Y. Fu C. Li N. Peng F. (2023). Sorcin regulate pyroptosis by interacting with NLRP3 inflammasomes to facilitate the progression of hepatocellular carcinoma. Cell. Death Dis. 14, 678. 10.1038/s41419-023-06096-1 37833249 PMC10575890

[B97] Li F. Zhang X. Q. Ho W. Tang M. Li Z. Bu L. (2023). mRNA lipid nanoparticle-mediated pyroptosis sensitizes immunologically cold tumors to checkpoint immunotherapy. Nat. Communications 14, 4223. 10.1038/s41467-023-39938-9 PMC10349854 37454146

[B98] Li P. Hong J. Liang C. Li Y. Gao L. Wu L. (2023). Endothelial cell-released extracellular vesicles trigger pyroptosis and vascular inflammation to induce atherosclerosis through the delivery of HIF1A-AS2. Faseb J. 37, e22942. 10.1096/fj.202201399rrr 37178006

[B99] Li G. Liu C. Yang L. Feng L. Zhang S. An J. (2023). Syringaresinol protects against diabetic nephropathy by inhibiting pyroptosis *via* NRF2-mediated antioxidant pathway. Cell. Biol. Toxicol. 39, 621–639. 10.1007/s10565-023-09790-0 36640193

[B100] Li L. Li Y. Lin J. Pang W. (2024). A pyroptosis-related gene signature predicts prognosis and tumor immune microenvironment in colorectal cancer. Technol. Cancer Res. Treat. 23, 15330338241277584. 10.1177/15330338241277584 39155627 PMC11331578

[B101] Li J. Zheng D. Qi S. Mao C. Jiang Y. (2025). Dual-functional nanosized albumin facilitates CRISPR-Cas9-PAR2 to mitigate cytokine storm *via* TLR4 and pyroptosis signaling. Small, 21 (4), 2405593. 10.1002/smll.202405593 39617988

[B102] Li Y. Cui Q. Zhou B. Zhang J. Guo R. Wang Y. (2024). RSAD2, a pyroptosis-related gene, predicts the prognosis and immunotherapy response for colorectal cancer. Am. J. Cancer Res. 14, 2507–2522. 10.62347/RGJO6884 38859852 PMC11162672

[B103] Li X. Zhang Z. Han Y. Zhang M. (2025a). NLRP3 inflammasome and pyroptosis: implications in inflammation and multisystem disorders. PeerJ 13, e19887. 10.7717/peerj.19887 40832584 PMC12360325

[B104] Li L. Xing Z. Wang J. Guo Y. Wu X. Ma Y. (2025b). Hyaluronic acid-mediated targeted nano-modulators for activation of pyroptosis for cancer therapy through multichannel regulation of Ca(2+) overload. Int. J. Biol. Macromol. 299, 140116. 10.1016/j.ijbiomac.2025.140116 39842602

[B105] Li X. Liu X. Zhou J. Zhang P. Chen S. Bai D. (2025c). Human dental follicle stem cell-derived exosomes reduce root resorption by inhibiting periodontal ligament cell pyroptosis. Stem Cell. Res. Ther. 16, 79. 10.1186/s13287-025-04216-6 39985080 PMC11846241

[B106] Li P. Yang X. Liu Q. Zhang H. Luo Z. (2025). Bladder cancer biomarker analysis and drugtarget prediction based on pyroptosis-related genes. Discov. Oncol. 16, 924. 10.1007/s12672-025-02754-2 40415077 PMC12104127

[B107] Li L. Huang H. C. He Y. Pang J. Y. Xiao S. C. Xia Z. F. (2025e). Cell-free DNA in sepsis: from molecular insights to clinical management. Mil. Med. Res. 12, 85. 10.1186/s40779-025-00668-2 41327452 PMC12670778

[B108] Li J. Ding B. Zheng P. Meng Q. Chen H. Tan J. (2026). Construction of diverse calcium-based nanomaterials through a microemulsion method for pyroptosis-initiated antitumor immunotherapy. Adv. Mater 38, e16225. 10.1002/adma.202516225 41085120

[B109] Liang F. Zhang F. Zhang L. Wei W. (2020). The advances in pyroptosis initiated by inflammasome in inflammatory and immune diseases. Inflamm. Res. 69, 159–166. 10.1007/s00011-020-01315-3 31932850

[B110] Liang D. Hu M. Tang Q. Huang M. Tang L. (2021). Nine pyroptosis-related lncRNAs are identified as biomarkers for predicting the prognosis and immunotherapy of endometrial carcinoma. Int. J. Gen. Med. 14, 8073–8085. 10.2147/ijgm.s338298 34803394 PMC8594792

[B111] Lin L. Zhang M. X. Zhang L. Zhang D. Li C. Li Y. L. (2021). Autophagy, pyroptosis, and ferroptosis: new regulatory mechanisms for atherosclerosis. Front. Cell. Dev. Biol. 9, 809955. 10.3389/fcell.2021.809955 35096837 PMC8793783

[B112] Lin J. Liu F. Gao F. Chen Y. Wang R. Wang X. (2022). Vesicular stomatitis virus sensitizes immunologically cold tumors to checkpoint blockade by inducing pyroptosis. Biochim. Biophys. Acta Mol. Basis Dis. 1868, 166538. 10.1016/j.bbadis.2022.166538 36096276

[B113] Liu S. Liang Q. (2025). Sepsis toxicity network reconstruction-dynamic signaling and multi-organ injury: a review. Biomol. Biomed. 26, 262–273. 10.17305/bb.2025.12931 40891647 PMC12505525

[B114] Liu X. Lieberman J. (2024). Inflammasome-independent pyroptosis. Curr. Opin. Immunol. 88, 102432. 10.1016/j.coi.2024.102432 38875738

[B115] Liu X. Luo P. Zhang W. Zhang S. Yang S. Hong F. (2023). Roles of pyroptosis in atherosclerosis pathogenesis. Biomed. Pharmacother. 166, 115369. 10.1016/j.biopha.2023.115369 37643484

[B116] Liu L. Cai Y. Deng C. (2023). Identification of ANXA3 as a biomarker associated with pyroptosis in ischemic stroke. Eur. J. Med. Res. 28, 596. 10.1186/s40001-023-01564-y 38102696 PMC10725036

[B117] Liu W. Peng J. Xiao M. Cai Y. Peng B. Zhang W. (2023). The implication of pyroptosis in cancer immunology: current advances and prospects. Genes. Dis. 10, 2339–2350. 10.1016/j.gendis.2022.04.019 37554215 PMC10404888

[B118] Liu Y. Li X. Sun T. Li T. Li Q. (2024). Pyroptosis in myocardial ischemia/reperfusion and its therapeutic implications. Eur. J. Pharmacol. 971, 176464. 10.1016/j.ejphar.2024.176464 38461908

[B119] Liu P. Yang S. Shao X. Li C. Wang Z. Dai H. (2024). Mesenchymal stem cells-derived exosomes alleviate acute lung injury by inhibiting alveolar macrophage pyroptosis. Stem Cells Transl. Med. 13, 371–386. 10.1093/stcltm/szad094 38349749 PMC11016849

[B120] Liu M. Chen J. Sun Y. (2025). Mechanistic insights into elevated caspase-8 expression driving caspase-3 activation and gasdermin E-dependent pyroptosis in alzheimer's disease. Exp. Cell. Res. 449, 114594. 10.1016/j.yexcr.2025.114594 40334809

[B121] Liu L. Li C. Liu Y. (2025). Epigenetic regulation of regulated cell death in aging-related diseases: clinical perspectives. Aging Dis. 10.14336/AD.2025.1098 PMC13653726 41213084

[B122] Liu B. Chen X. Zhen Z. Li J. Zheng P. Feng L. (2025). Nanospiky copper peroxide as lactate-modulating nanoadjuvant to induce cuproptosis and pyroptosis for enhanced tumor immunotherapy. Nano Lett. 25, 15686–15697. 10.1021/acs.nanolett.5c04293 41090566

[B123] Liu Y. Zhou J. Song M. Wang J. Shang Q. Du J. (2026a). CTHRC1 drives megakaryocyte-mediated immunotherapy resistance in esophageal squamous cell carcinoma via the integrin αIIbβ3/Rap1 pathway and is targetable by mRNA vaccination. Molecular cancer 25, 178. 10.1186/s12943-026-02670-1 PMC13377830 42169077

[B124] Liu Y. Zhou J. Gong Z. Feng Y. Li X. Shang Q. (2026b). Mapping the Global Research Landscape of Nanomaterial-based Cancer Vaccines: A Comprehensive Bibliometric and Visualized Review.

[B125] Liu Q. Shi Y. Qin L. (2026). Targeting pyroptosis in atherosclerosis: emerging pharmacologic strategies and natural compound-based therapeutics-a narrative review. Int. J. Clin. Pharm. 48, 67–79. 10.1007/s11096-025-02065-0 41369784

[B126] Luo J. Liao Z. Zhang S. (2026). Mechanistic insights and therapeutic potential of small nucleolar RNA host genes in the carcinogenesis of hepatocellular carcinoma. Cancer Med. 15, e71460. 10.1002/cam4.71460 41474641 PMC12755402

[B127] Ma M. Wang F. Cui P. Zhang Y. Ma H. He Y. (2025). RIOK2 kinase regulates the translocation of the FADD-RIPK1-Caspase-8 complex to the ER and the cleavage of Gasdermin D to drive pyroptosis. Nat. Commun. 16, 10060. 10.1038/s41467-025-65012-7 41249793 PMC12623739

[B128] Nadjar J. Monnier S. Bastien E. Huber A. L. Oddou C. Bardoulet L. (2024). Optogenetically controlled inflammasome activation demonstrates two phases of cell swelling during pyroptosis. Sci. Signal 17, eabn8003. 10.1126/scisignal.abn8003 38652763

[B129] Najafi S. Rahimpour A. Ahmadieh H. Tehrani M. M. Khalilzad M. A. Suri F. (2024). The significance of chemical transfection/transduction enhancers in promoting the viral vectors-assisted gene delivery approaches: a focus on potentials for inherited retinal diseases. Electron. J. Biotechnol. 72, 29–40. 10.1016/j.ejbt.2024.07.005

[B130] Ngai W. S. C. Yang S. Zeng X. Liu Y. Lin F. Wang X. (2022). Bioorthogonally activatable base editing for On-Demand pyroptosis. J. Am. Chem. Soc. 144, 5411–5417. 10.1021/jacs.1c12924 35290047

[B131] Ning W. Acharya A. Li S. Schmalz G. Huang S. (2022). Identification of key pyroptosis-related genes and distinct pyroptosis-related clusters in periodontitis. Front. Immunol. 13, 862049. 10.3389/fimmu.2022.862049 35844512 PMC9281553

[B132] Niu Q. Liu Y. Zheng Y. Tang Z. Qian Y. Qi R. (2022). Co-delivery of nigericin and decitabine using hexahistidine-metal nanocarriers for pyroptosis-induced immunotherapeutics. Acta Pharm. Sin. B 12, 4458–4471. 10.1016/j.apsb.2022.11.002 36562000 PMC9764131

[B133] Niu J. Wen Y. Zhang F. Li S. Miao J. Liang H. (2024). Pericentriolar material 1 promotes intestinal inflammation in ulcerative colitis by activating NLRP3/gasdermin D-mediated macrophage pyroptosis. Faseb J. 38, e70054. 10.1096/fj.202401185r 39297783

[B134] Oh C. Spears T. J. Aachoui Y. (2025). Inflammasome-mediated pyroptosis in defense against pathogenic bacteria. Immunol. Rev. 329, e13408. 10.1111/imr.13408 39404258 PMC11741929

[B135] Oladapo A. Jackson T. Menolascino J. Periyasamy P. (2024). Role of pyroptosis in the pathogenesis of various neurological diseases. Brain Behav. Immun. 117, 428–446. 10.1016/j.bbi.2024.02.001 38336022 PMC10911058

[B136] Ouyang X. Zhou J. Lin L. Zhang Z. Luo S. Hu D. (2023). Pyroptosis, inflammasome, and gasdermins in tumor immunity. Innate Immun. 29, 3–13. 10.1177/17534259221143216 36632024 PMC10164276

[B137] Ouyang B. Shan C. Shen S. Dai X. Chen Q. Su X. (2024). AI-powered omics-based drug pair discovery for pyroptosis therapy targeting triple-negative breast cancer. Nat. Commun. 15, 7560. 10.1038/s41467-024-51980-9 39215014 PMC11364624

[B138] Pan Z. Xu K. Huang G. Hu H. Yang H. Shen H. (2024). Pyroptotic-Spatiotemporally selective delivery of siRNA against pyroptosis and autoimmune diseases. Adv. Mater 36, e2407115. 10.1002/adma.202407115 39081086

[B139] Park J. Rhoo K. Y. Kim Y. Kim Y. S. Paik S. R. (2025). Cell-Division-Independent rapid expression of DNA delivered with α-Synuclein-Gold nanoparticle conjugates. ACS Appl. Mater Interfaces 17, 14846–14858. 10.1021/acsami.4c17967 40014054

[B140] Pérez-Rodríguez F. Mercanoglu Taban B. (2019). A state-of-art review on multi-drug resistant pathogens in foods of animal origin: risk factors and mitigation strategies. Front. Microbiology 10, 2091. 10.3389/fmicb.2019.02091 31555256 PMC6742700

[B141] Phungphong S. Suthivanich P. Boonhoh W. Punsawad C. Cheng Z. Bupha-Intr T. (2025). Targeting NLRP3 inflammasome attenuates cardiac pyroptosis and fibrosis in estrogen-deficient diabetic rats. Pflugers Arch. 477, 935–952. 10.1007/s00424-025-03092-6 40383837

[B142] Qi F. Guo Y. Xu Y. Liu Y. Hu P. Shi J. (2025). Calcium ion nanomodulators induce pyroptosis for tumor immunotherapy. Nanoscale 17, 25516–25525. 10.1039/d5nr01897h 41159427

[B143] Qiu Y. Huang Y. Chen M. Yang Y. Li X. Zhang W. (2022). Mitochondrial DNA in NLRP3 inflammasome activation. Int. Immunopharmacol. 108, 108719. 10.1016/j.intimp.2022.108719 35349960

[B144] Raneros A. B. Bernet C. R. Flórez A. B. Suarez-Alvarez B. (2021). An epigenetic insight into NLRP3 inflammasome activation in inflammation-related processes. Biomedicines 9, 1614. 10.3390/biomedicines9111614 34829842 PMC8615487

[B145] Ren Y. Feng M. Hao X. Liu X. Li J. Li P. (2023). USP48 stabilizes gasdermin E to promote pyroptosis in cancer. Cancer Res. 83, 1074–1093. 10.1158/0008-5472.can-22-1812 36607699

[B146] Ren C. Chen J. Che Q. Jia Q. Lu H. Qi X. (2023). IL-37 alleviates TNF-α-induced pyroptosis of rheumatoid arthritis fibroblast-like synoviocytes by inhibiting the NF-κB/GSDMD signaling pathway. Immunobiology 228, 152382. 10.1016/j.imbio.2023.152382 37075579

[B147] Ridker P. M. Everett B. M. Thuren T. MacFadyen J. G. Chang W. H. Ballantyne C. (2017a). Antiinflammatory therapy with canakinumab for atherosclerotic disease. N. Engl. Journal Medicine 377, 1119–1131. 10.1056/nejmoa1707914 28845751

[B148] Ridker P. M. MacFadyen J. G. Thuren T. Everett B. M. Libby P. Glynn R. J. (2017b). Effect of interleukin-1β inhibition with canakinumab on incident lung cancer in patients with atherosclerosis: exploratory results from a randomised, double-blind, placebo-controlled trial. Lancet London, Engl. 390, 1833–1842. 10.1016/s0140-6736(17)32247-x 28855077

[B149] Ruan J. Xia Y. Ma Y. Xu X. Luo S. Yi J. (2025). Milk-derived exosomes as functional nanocarriers in wound healing: mechanisms, applications, and future directions. Mater. Today. Bio 32, 101715. 10.1016/j.mtbio.2025.101715 PMC12003018 40242483

[B150] Salehnia Z. Rezaee D. Ehtiati S. Bakhtiari M. Khalilzad M. A. Najafi S. (2026). Liquid biopsy for the management of gastrointestinal cancers. Clin. Chimica Acta; International Journal Clinical Chemistry 578, 120474. 10.1016/j.cca.2025.120474 40652998

[B151] Seo W. Jung B. Roh T. Shin H. J. Song I. C. Chung C. (2025). Inflammasomes and pyroptosis in cancer: mechanisms and therapeutic advances. J. Hematol. Oncol. 18, 113. 10.1186/s13045-025-01763-6 41291897 PMC12751675

[B152] Shang J. Liu L. Zhang J. Li B. Zhang P. Wang H. (2025). Engineering a targeted daphnetin delivery system to enhance spinal cord injury treatment effectiveness. Mater Today Bio 35, 102576. 10.1016/j.mtbio.2025.102576 PMC12689221 41377580

[B153] Shao F. (2021). Gasdermins: making pores for pyroptosis. Nat. Rev. Immunol. 21, 620–621. 10.1038/s41577-021-00602-2 34580452

[B154] Shao B. Z. Wang S. L. Pan P. Yao J. Wu K. Li Z. S. (2019). Targeting NLRP3 inflammasome in inflammatory bowel disease: putting out the fire of inflammation. Inflammation 42, 1147–1159. 10.1007/s10753-019-01008-y 30937839

[B155] Shen H. Peng Y. Xie Q. Ren Y. Hu J. Qin P. (2025). Prognostic model construction and immune microenvironment analysis of pyroptosis-related genes in hepatocellular carcinoma based on single-cell RNA sequencing. Front. Immunology 16, 1595539. 10.3389/fimmu.2025.1595539 PMC12408283 40918134

[B156] Shi C. Cao P. Wang Y. Zhang Q. Zhang D. Wang Y. (2023). PANoptosis: a cell death characterized by pyroptosis, apoptosis, and necroptosis. J. Inflamm. Res. 16, 1523–1532. 10.2147/jir.s403819 37077221 PMC10106823

[B157] Shoeib H. M. Keshk W. A. Al-Ghazaly G. M. Wagih A. A. El-Dardiry S. A. (2023). Interplay between long non-coding RNA MALAT1 and pyroptosis in diabetic nephropathy patients. Gene 851, 146978. 10.1016/j.gene.2022.146978 36270460

[B158] Sollberger G. (2022). Approaching neutrophil pyroptosis. J. Mol. Biol. 434, 167335. 10.1016/j.jmb.2021.167335 34757055

[B159] Song S. Guo R. Mehmood A. Zhang L. Yin B. Yuan C. (2022). Liraglutide attenuate central nervous inflammation and demyelination through AMPK and pyroptosis-related NLRP3 pathway. CNS Neurosci. Ther. 28, 422–434. 10.1111/cns.13791 34985189 PMC8841291

[B160] Song Y. Peng Y. Wang B. Zhou X. Cai Y. Chen H. (2024). The roles of pyroptosis in the pathogenesis of autoimmune diseases. Life Sci. 359, 123232. 10.1016/j.lfs.2024.123232 39537097

[B161] Song K. Wu Y. Tan S. (2025). Caspases in PANoptosis. Curr. Res. Transl. Med. 73, 103502. 10.1016/j.retram.2025.103502 39985853

[B162] Song A. Yang Q. C. Wang W. D. Wang S. Li H. Wu L. (2025). Tumor-Intrinsic SIRPA drives pyroptosis evasion in head and neck cancer. J. Dent. Res. 104, 645–655. 10.1177/00220345241305590 39904995

[B163] Sordi M. B. Magini R. S. Panahipour L. Gruber R. (2021). Pyroptosis-Mediated periodontal disease. Int. J. Mol. Sci. 23, 372. 10.3390/ijms23010372 35008798 PMC8745163

[B164] Stoess C. Leszczynska A. Kui L. Feldstein A. E. (2023). Pyroptosis and gasdermins-emerging insights and therapeutic opportunities in metabolic dysfunction-associated steatohepatitis. Front. Cell. Dev. Biol. 11, 1218807. 10.3389/fcell.2023.1218807 37664463 PMC10470644

[B165] Sun S. W. Tong W. J. Zheng G. Q. Tuo Q. H. Lei X. Y. Liao D. F. (2021). Pyroptotic cell-derived microparticle: an atherogenic factor in infectious diseases. Med. Hypotheses 146, 110370. 10.1016/j.mehy.2020.110370 33308934

[B166] Sun P. Wang X. Zhong J. Yu D. Xuan H. Xu T. (2023). Development and validation of a pyroptosis-related genes signature for risk stratification in gliomas. Front. Genetics 14, 1087563. 10.3389/fgene.2023.1087563 PMC9968976 36861130

[B167] Sun P. Chen X. Wang Y. Wang X. Li K. Song H. (2025). Regulatory mechanisms of luteolin in inflammatory respiratory diseases. Front. Pharmacol. 16, 1720824. 10.3389/fphar.2025.1720824 41394141 PMC12696170

[B168] Taha S. R. Karimi M. Mahdavi B. Yousefi Tehrani M. Bemani A. Kabirian S. (2025). Crosstalk between non-coding RNAs and programmed cell death in colorectal cancer: implications for targeted therapy. Epigenetics Chromatin 18, 3. 10.1186/s13072-024-00560-8 39810224 PMC11734566

[B169] Tan Y. Chen Q. Li X. Zeng Z. Xiong W. Li G. (2021). Pyroptosis: a new paradigm of cell death for fighting against cancer. J. Exp. Clin. Cancer Res. 40, 153. 10.1186/s13046-021-01959-x 33941231 PMC8091792

[B170] Tan J. Q. Li Z. Chen G. Wu M. Feng J. L. Kong S. Y. (2022). The natural compound from Garcinia bracteata mainly induces GSDME-Mediated pyroptosis in esophageal cancer cells. Phytomedicine 102, 154142. 10.1016/j.phymed.2022.154142 35623158

[B171] Tan X. Yang J. Li Y. Wan K. Feng S. Jing X. (2025). Development of Z(HPV16E7)- granzyme B immunoaffitoxin: dual mechanisms targeting hpv16-positive cervical cancer through epithelial-mesenchymal transition inhibition and cell death. Front. Immunol. 16, 1616715. 10.3389/fimmu.2025.1616715 40761801 PMC12319584

[B172] Tang X. Guo J. Qi F. Rezaei M. J. (2024). Role of non-coding RNAs and exosomal non-coding RNAs in vasculitis: a narrative review. Int. J. Biol. Macromol. 261, 129658. 10.1016/j.ijbiomac.2024.129658 38266857

[B173] Tian J. Gao M. Zhu J. Xu H. Ji H. Xia D. (2024). Platelets camouflaged nanovehicle improved bladder cancer immunotherapy by triggering pyroptosis. Theranostics 14, 6692–6707. 10.7150/thno.99040 39479459 PMC11519802

[B174] Trois C. Del Fabro L. D. Baulin V. A. (2025). Machine learning unveils large-scale impact of Posidonia oceanica on Mediterranean Sea water. Sci. Total Environ. 968, 178802. 10.1016/j.scitotenv.2025.178802 39987821

[B175] Tsuchiya K. (2020). Inflammasome-associated cell death: pyroptosis, apoptosis, and physiological implications. Microbiol. Immunol. 64, 252–269. 10.1111/1348-0421.12771 31912554

[B176] Tuncer M. Alcan S. (2023). Pyroptosis: a new therapeutic strategy in cancer. Mol. Biol. Rep. 50, 6191–6200. 10.1007/s11033-023-08482-6 37243815

[B177] Wang Q. Wu J. Zeng Y. Chen K. Wang C. Yang S. (2020). Pyroptosis: a pro-inflammatory type of cell death in cardiovascular disease. Clin. Chim. Acta 510, 62–72. 10.1016/j.cca.2020.06.044 32622968

[B178] Wang L. Qin X. Liang J. Ge P. (2021). Induction of pyroptosis: a promising strategy for cancer treatment. Front. Oncol. 11, 635774. 10.3389/fonc.2021.635774 33718226 PMC7953901

[B179] Wang Y. Karki R. Zheng M. Kancharana B. Lee S. Kesavardhana S. (2021). Cutting edge: Caspase-8 is a linchpin in Caspase-3 and gasdermin D activation to control cell death, cytokine release, and host defense during influenza A virus infection. J. Immunol. 207, 2411–2416. 10.4049/jimmunol.2100757 34663620 PMC8853633

[B180] Wang Z. Yu H. Zhuang W. Chen J. Jiang Y. Guo Z. (2022). Cell pyroptosis in picornavirus and its potential for treating viral infection. J. Med. Virol. 94, 3570–3580. 10.1002/jmv.27813 35474513

[B181] Wang J. Huangfu M. Li X. Han M. Liu G. Yu D. (2022). Osthole induces apoptosis and Caspase-3/GSDME-Dependent pyroptosis *via* NQO1-Mediated ROS generation in HeLa cells. Oxid. Med. Cell. Longev. 2022, 8585598. 10.1155/2022/8585598 35720178 PMC9200556

[B182] Wang W. Zhang L. Sun Z. (2022). Eliciting pyroptosis to fuel cancer immunotherapy: mechanisms and strategies. Cancer Biol. Med. 19, 948–964. 10.20892/j.issn.2095-3941.2022.0049 35856558 PMC9334758

[B183] Wang Y. Ding L. Wang R. Guo Y. Yang Z. Yu L. (2022). Circ_0004951 promotes pyroptosis of renal tubular cells *via* the NLRP3 inflammasome in diabetic kidney disease. Front. Med. (Lausanne) 9, 828240. 10.3389/fmed.2022.828240 35733856 PMC9207212

[B184] Wang S. Moreau F. Chadee K. (2022). The colonic pathogen Entamoeba histolytica activates caspase-4/1 that cleaves the pore-forming protein gasdermin D to regulate IL-1β secretion. PLoS Pathog. 18, e1010415. 10.1371/journal.ppat.1010415 35303042 PMC8967020

[B185] Wang H. G. Wang D. Sarfraz M. Afzal A. Jing M. R. Zhang Y. X. (2023). Endogenous hydrogen sulfide inhibition suppresses tumor growth by promoting apoptosis and pyroptosis in esophageal cancer cells. Transl. Oncol. 38, 101770. 10.1016/j.tranon.2023.101770 37716259 PMC10514559

[B186] Wang J. Sun H. Guo R. Guo J. Tian X. Wang J. (2023). Exosomal miR-23b-3p from bone mesenchymal stem cells alleviates experimental autoimmune encephalomyelitis by inhibiting microglial pyroptosis. Exp. Neurol. 363, 114374. 10.1016/j.expneurol.2023.114374 36907352

[B187] Wang N. Liu C. Li Y. Huang D. Wu X. Kou X. (2023). A cooperative nano-CRISPR scaffold potentiates immunotherapy *via* activation of tumour-intrinsic pyroptosis. Nat. Commun. 14, 779. 10.1038/s41467-023-36550-9 36774382 PMC9922300

[B188] Wang X. He S. Cheng P. Pu K. (2023). A dual-locked tandem fluorescent probe for imaging of pyroptosis in cancer chemo-immunotherapy. Adv. Mater 35, e2206510. 10.1002/adma.202206510 36317605

[B189] Wang Y. Niu B. Tian Y. Lan H. Zhou Z. Li Y. (2024). Mitoxantrone combined with engineered TRAIL-Nanovesicles for enhanced cancer immunotherapy *via* converting apoptosis into pyroptosis. Adv. Healthc. Mater 13, e2401723. 10.1002/adhm.202401723 39049538

[B190] Wang J. B. Gao Y. X. Ye Y. H. Zheng Q. L. Luo H. Y. Wang S. H. (2024). Comprehensive multi-omics analysis of pyroptosis for optimizing neoadjuvant immunotherapy in patients with gastric cancer. Theranostics 14, 2915–2933. 10.7150/thno.93124 38773976 PMC11103507

[B191] Wang P. Wang Z. Lin Y. Castellano L. Stebbing J. Zhu L. (2024). Development of a novel pyroptosis-associated lncRNA biomarker signature in lung adenocarcinoma. Mol. Biotechnol. 66, 332–353. 10.1007/s12033-023-00757-4 37154865

[B192] Wang S. Zhang J. Chen J. Tang L. Ke M. Xue Y. (2024). ω-3PUFAs inhibit hypoxia-induced retinal neovascularization *via* regulating microglial pyroptosis through METTL14-Mediated m6A modification of IFNB1 mRNA. Appl. Biochem. Biotechnol. 196, 5936–5952. 10.1007/s12010-023-04795-1 38175416

[B193] Wang H. Wang T. Yan S. Tang J. Zhang Y. Wang L. (2024). Crosstalk of pyroptosis and cytokine in the tumor microenvironment: from mechanisms to clinical implication. Mol. Cancer 23, 268. 10.1186/s12943-024-02183-9 39614288 PMC11607834

[B194] Wang J. Wu Z. Zhu M. Zhao Y. Xie J. (2024). ROS induced pyroptosis in inflammatory disease and cancer. Front. Immunol. 15, 1378990. 10.3389/fimmu.2024.1378990 39011036 PMC11246884

[B195] Wang Y. P. Liu X. Xu X. D. Hui L. Xu G. H. Ma M. Q. (2025). LncRNA MIR17HG accelerates the development of atherosclerosis by promoting macrophage pyroptosis through the miR-301a-3p/TXNIP pathway. Int. Immunopharmacol. 164, 115407. 10.1016/j.intimp.2025.115407 40854265

[B196] Wang J. Li J. He X. Zhang H. Zheng X. Luo H. (2025). Exosomal miRNA-25-3p from platelet-rich plasma alleviates chondrocyte pyroptosis and knee osteoarthritis by regulating MAP4K2/Hippo/TNFAIP3 pathway. Funct. Integr. Genomics 25, 268. 10.1007/s10142-025-01780-1 41354766

[B197] Wang Y. Cui Y. Liu Z. Liu L. Huang Z. Wang Q. (2025). Acevaltrate overcomes myeloma resistance to bortezomib *via* pyroptosis by promoting BAX translocalization to mitochondria. Eur. J. Pharmacol. 996, 177572. 10.1016/j.ejphar.2025.177572 40180268

[B198] Wang S. Kong L. Wang L. Zhuang Y. Guo C. Zhang Y. (2025). Viral expression of NE/PPE enhances anti-colorectal cancer efficacy of oncolytic adenovirus by promoting TAM M1 polarization to reverse insufficient effector memory/effector CD8(+) T cell infiltration. J. Exp. Clin. Cancer Res. 44, 97. 10.1186/s13046-025-03358-y 40082916 PMC11907943

[B199] Wang M. Chen D. Zhang D. Han H. Xie M. Zhang Y. (2026). Granzyme B knockdown inhibits NLRP3-mediated pyroptosis and the JAK2/STAT3 signaling in renal fibrosis. Tissue Cell. 99, 103216. 10.1016/j.tice.2025.103216 41447776

[B200] Wang H. Wu L. Liu C. Zhao X. Cui L. Gao J. (2026). A novel lncRNA, lncMCL1, modulates neural pyroptosis associated with epilepsy *via* stabilizing DDX3X. Cell. Death Differ. 33, 374–391. 10.1038/s41418-025-01584-7 40983632 PMC12881509

[B201] Wei X. Ran S. Yan X. Huang J. Xue L. He T. C. (2025). Pyroptosis in pulpitis. J. Inflamm. Res. 18, 5867–5879. 10.2147/jir.s516502 40322528 PMC12050040

[B202] Wen R. Liu Y. P. Tong X. X. Zhang T. N. Yang N. (2022). Molecular mechanisms and functions of pyroptosis in sepsis and sepsis-associated organ dysfunction. Front. Cell. Infect. Microbiol. 12, 962139. 10.3389/fcimb.2022.962139 35967871 PMC9372372

[B203] Wen J. Xuan B. Liu Y. Wang L. He L. Meng X. (2023). NLRP3 inflammasome-induced pyroptosis in digestive system tumors. Front. Immunol. 14, 1074606. 10.3389/fimmu.2023.1074606 37081882 PMC10110858

[B204] Wright S. S. Vasudevan S. O. Rathinam V. A. (2022). Mechanisms and consequences of noncanonical inflammasome-mediated pyroptosis. J. Mol. Biol. 434, 167245. 10.1016/j.jmb.2021.167245 34537239 PMC8844060

[B205] Wu Q. Ji Z. (2026). Pyroptosis in acute pancreatitis: from pathogenesis to treatment. Int. J. Surg. 112, 4657–4671. 10.1097/js9.0000000000003932 41247814

[B206] Wu J. J. Cai J. Tang Y. Lu B. (2023). The noncanonical inflammasome-induced pyroptosis and septic shock. Semin. Immunol. 70, 101844. 10.1016/j.smim.2023.101844 37778179

[B207] Wu J. Sun J. Meng X. (2021a). Pyroptosis by caspase-11 inflammasome-gasdermin D pathway in autoimmune diseases. Pharmacol. Res. 165, 105408. 10.1016/j.phrs.2020.105408 33412278

[B208] Wu J. Zhu Y. Luo M. Li L. (2021b). Comprehensive analysis of pyroptosis-related genes and tumor microenvironment infiltration characterization in breast cancer. Front. Immunol. 12, 748221. 10.3389/fimmu.2021.748221 34659246 PMC8515898

[B209] Wu J. Wang L. Xu J. (2022). The role of pyroptosis in modulating the tumor immune microenvironment. Biomark. Res. 10, 45. 10.1186/s40364-022-00391-3 35739593 PMC9229852

[B210] Wu X. Wan T. Gao X. Fu M. Duan Y. Shen X. (2022). Microglia pyroptosis: a candidate target for neurological diseases treatment. Front. Neurosci. 16, 922331. 10.3389/fnins.2022.922331 35937897 PMC9354884

[B211] Wu H. Qian D. Bai X. Sun S. (2022). Targeted pyroptosis is a potential therapeutic strategy for cancer. J. Oncol. 2022, 2515525. 10.1155/2022/2515525 36467499 PMC9715319

[B212] Wu F. Wang M. Zhong T. Xiao C. Chen X. Huang Y. (2023). Inhibition of CDC20 potentiates anti-tumor immunity through facilitating GSDME-Mediated pyroptosis in prostate cancer. Exp. Hematol. Oncol. 12, 67. 10.1186/s40164-023-00428-9 37528490 PMC10391908

[B213] Wu Q. Guan Y. B. Zhang K. J. Li L. Zhou Y. (2023). Tanshinone IIA mediates protection from diabetes kidney disease by inhibiting oxidative stress induced pyroptosis. J. Ethnopharmacol. 316, 116667. 10.1016/j.jep.2023.116667 37257702

[B214] Wu G. Chen B. Jiang J. Chen Y. Chen Y. Wang H. (2023). Identification of a pyroptosis-based model for predicting clinical outcomes from immunotherapy in patients with metastatic melanoma. Cancer Med. 12, 4921–4937. 10.1002/cam4.5178 36151761 PMC9972144

[B215] Wu S. Yao L. Zhang W. Chen P. Jiang J. Ma Y. (2025). Bioinformatics analysis and validation of novel biomarkers and competitive endogenous RNA networks involved in pyroptosis in diabetic nephropathy. Sci. Rep. 15, 5530. 10.1038/s41598-025-87854-3 39953123 PMC11829041

[B216] Wu Y. Zhao Z. Ma M. Zhang W. Liu W. Liang X. (2025). Ultrasound-activated erythrocyte membrane-camouflaged Pt (II) layered double hydroxide enhances PD-1 inhibitor efficacy in triple-negative breast cancer through cGAS-STING pathway-mediated immunogenic cell death. Theranostics 15, 1456–1477. 10.7150/thno.102284 39816689 PMC11729553

[B217] Xia L. Yang M. Zang N. Song J. Chen J. Hu H. (2024). PEGylated β-Cell-Targeting exosomes from mesenchymal stem cells improve β cell function and quantity by suppressing NRF2-Mediated ferroptosis. Int. J. Nanomedicine 19, 9575–9596. 10.2147/ijn.s459077 39296939 PMC11410040

[B218] Xian Y. Wang X. Yu Y. Chen X. (2024). Transcriptomics confirms IRF1 as a key regulator of pyroptosis in diabetic retinopathy. Biochem. Biophys. Res. Commun. 709, 149760. 10.1016/j.bbrc.2024.149760 38554602

[B219] Xiao Y. Zhang T. Ma X. Yang Q. C. Yang L. L. Yang S. C. (2021). Microenvironment-responsive prodrug-induced pyroptosis boosts cancer immunotherapy. Adv. Sci. (Weinh) 8, e2101840. 10.1002/advs.202101840 34705343 PMC8693073

[B220] Xie J. Peng B. Xiao Y. Chen X. Zhang X. Chen D. (2025). Overcoming cancer immunotherapy barriers *via* nanomaterial-mediated pyroptosis. J. Mater Chem. B 13, 11485–11507. 10.1039/d5tb01024a 40856020

[B221] Xin P. Tan Z. Wang Z. Chen Y. Zhuang Y. (2024). Circular RNA hsa_circ_0000175 serves as a potential biomarker for rheumatoid arthritis *via* miR-31-5p/GSDME axis. Biochem. Genet. 62, 2522–2539. 10.1007/s10528-023-10576-6 37968534

[B222] Xing S. S. Yang J. Li W. J. Li J. Chen L. Yang Y. T. (2020). Salidroside decreases atherosclerosis plaque formation *via* inhibiting endothelial cell pyroptosis. Inflammation 43, 433–440. 10.1007/s10753-019-01106-x 32076940

[B223] Xu K. Yang H. Fang J. Qiu K. Shen H. Huang G. (2024). Self-adaptive pyroptosis-responsive nanoliposomes block pyroptosis in autoimmune inflammatory diseases. Bioact. Mater 36, 272–286. 10.1016/j.bioactmat.2024.02.022 38496034 PMC10940868

[B224] Xu L. Li S. Qi J. Mi Y. Zhang Y. Yang Y. (2025). Effusol ameliorates ischemic stroke by targeting NLRP3 protein to regulate NLRP3 inflammasome-mediated pyroptosis. Phytomedicine 136, 156253. 10.1016/j.phymed.2024.156253 39615210

[B225] Xu G. Ma G. Sun J. Yu X. Sun J. Gao B. (2025). Comprehensive analysis of pyroptosis-related genes in psoriasis and targeted gene editing of CASP1 and CASP5 using lipid nanoparticles to alleviate skin inflammation. Front. Bioeng. Biotechnol. 13, 1639869. 10.3389/fbioe.2025.1639869 40771724 PMC12325318

[B226] Yan B. Zhang Y. Liang C. Liu B. Ding F. Wang Y. (2020). Stem cell-derived exosomes prevent pyroptosis and repair ischemic muscle injury through a novel exosome/circHIPK3/FOXO3a pathway. Theranostics 10, 6728–6742. 10.7150/thno.42259 32550900 PMC7295049

[B227] Yan M. Li W. Wei R. Li S. Liu Y. Huang Y. (2023). Identification of pyroptosis-related genes and potential drugs in diabetic nephropathy. J. Transl. Med. 21, 490. 10.1186/s12967-023-04350-w 37480090 PMC10360355

[B228] Yang G. Lu C. Mei Z. Sun X. Han J. Qian J. (2021). Association of cancer stem cell radio-resistance under ultra-high dose rate FLASH irradiation with lysosome-mediated autophagy. Front. Cell. Dev. Biol. 9, 672693. 10.3389/fcell.2021.672693 33996830 PMC8116574

[B229] Yang F. Bettadapura S. N. Smeltzer M. S. Zhu H. Wang S. (2022). Pyroptosis and pyroptosis-inducing cancer drugs. Acta Pharmacol. Sin. 43, 2462–2473. 10.1038/s41401-022-00887-6 35288674 PMC9525650

[B230] Yang Q. Ma X. Xiao Y. Zhang T. Yang L. Yang S. (2022). Engineering prodrug nanomicelles as pyroptosis inducer for codelivery of PI3K/mTOR and CDK inhibitors to enhance antitumor immunity. Acta Pharm. Sin. B 12, 3139–3155. 10.1016/j.apsb.2022.02.024 35865097 PMC9293721

[B231] Yang Y. Yang J. Zhu N. Qiu H. Feng W. Chen Y. (2023). Tumor-targeting hydroxyapatite nanoparticles for remodeling tumor immune microenvironment (TIME) by activating mitoDNA-pyroptosis pathway in cancer. J. Nanobiotechnology 21, 470. 10.1186/s12951-023-02231-4 38062467 PMC10704647

[B232] Yang H. Zhang Y. Du Z. Wu T. Yang C. (2023). Hair follicle mesenchymal stem cell exosomal lncRNA H19 inhibited NLRP3 pyroptosis to promote diabetic mouse skin wound healing. Aging (Albany NY) 15, 791–809. 10.18632/aging.204513 36787444 PMC9970314

[B233] Yang X. Cui X. Wang G. Zhou M. Wu Y. Du Y. (2024). HDAC inhibitor regulates the tumor immune microenvironment *via* pyroptosis in triple negative breast cancer. Mol. Carcinog. 63, 1800–1813. 10.1002/mc.23773 38860600

[B234] Yang T. An J. Xu X. Li B. Dou Z. (2025). Research progress on pyroptosis regulated by mesenchymal stem cell-derived exosomes. Front. Immunol. 16, 1717465. 10.3389/fimmu.2025.1717465 41425572 PMC12711477

[B235] Yang J. Yang J. Liu K. Wang S. Hu Y. Li J. (2026). Microfluidic-assisted metal-polyphenol cloaked bacteria enable CRISPR/dCas9-Mediated pyroptosis Cascade amplification for effective tumor immunotherapy. Adv. Healthcare Materials 15, e04837. 10.1002/adhm.202504837 41549804

[B236] Yao Y. Wang Z. Li J. Peng A. Cao Y. Liang N. (2024). Pyroptosis and its role in autoimmune skin disease. Exp. Dermatol 33, e15135. 10.1111/exd.15135 39021278

[B237] Yao X. Ji J. Chen D. Zhu Y. Cai X. (2025). EZH2-induced histone methylation in the Nrf2 promoter region mediates pyroptosis in inflammatory cardiomyocyte injury. Biochim. Biophys. Acta Gen. Subj. 1869, 130799. 10.1016/j.bbagen.2025.130799 40157552

[B238] Ye L. Wang L. Kuang G. Zhang Z. Peng Q. He M. (2025). IL-27 aggravates acute hepatic injury by promoting macrophage M1 polarization to induce Caspase-11 mediated Pyroptosis *in vitro* and *in vivo* . Cytokine 188, 156881. 10.1016/j.cyto.2025.156881 39913960

[B239] Yin H. Chen T. Hu X. Zhu W. Li Y. Sun W. (2024). Pyroptosis-inducing biomaterials pave the way for transformative antitumor immunotherapy. Adv. Sci. (Weinh) 11, e2410336. 10.1002/advs.202410336 39501932 PMC11653674

[B240] Yin Q. Song S. Y. Bian Y. Wang Y. Deng A. Lv J. (2024). Unlocking the potential of pyroptosis in tumor immunotherapy: a new horizon in cancer treatment. Front. Immunol. 15, 1381778. 10.3389/fimmu.2024.1381778 38947336 PMC11211258

[B241] You R. He X. Zeng Z. Zhan Y. Xiao Y. Xiao R. (2022). Pyroptosis and its role in autoimmune disease: a potential therapeutic target. Front. Immunol. 13, 841732. 10.3389/fimmu.2022.841732 35693810 PMC9174462

[B242] Yu P. Zhang X. Liu N. Tang L. Peng C. Chen X. (2021). Pyroptosis: mechanisms and diseases. Signal Transduct. Target Ther. 6, 128. 10.1038/s41392-021-00507-5 33776057 PMC8005494

[B243] Yu S. Zhang Y. Zhou Z. Li J. Meng H. (2025). Nanomaterials-induced pyroptosis: advancing novel therapeutic pathways in nanomedicine. Small Methods 9, e2401290. 10.1002/smtd.202401290 39853957

[B244] Yuan Z. Jiang G. Yuan Y. Liang Q. Hou Y. Zhang W. (2025). 5-FU@HFn combined with decitabine induces pyroptosis and enhances antitumor immunotherapy for chronic myeloid leukemia. J. Nanobiotechnology 23, 252. 10.1186/s12951-025-03335-9 40148810 PMC11951746

[B245] Yue R. Lu S. Luo Y. Zeng J. Liang H. Qin D. (2022). Mesenchymal stem cell-derived exosomal microRNA-182-5p alleviates myocardial ischemia/reperfusion injury by targeting GSDMD in mice. Cell. Death Discov. 8, 202. 10.1038/s41420-022-00909-6 35422485 PMC9010441

[B246] Zeng Y. Cai Y. Chai P. Mao Y. Chen Y. Wang L. (2022). Optimization of cancer immunotherapy through pyroptosis: a pyroptosis-related signature predicts survival benefit and potential synergy for immunotherapy in glioma. Front. Immunol. 13, 961933. 10.3389/fimmu.2022.961933 35990696 PMC9382657

[B247] Zhang J. Zhang J. Zhang Y. Liu W. Ni W. Huang X. (2020). Mesenchymal stem cells-derived exosomes ameliorate intervertebral disc degeneration through inhibiting pyroptosis. J. Cell. Mol. Med. 24, 11742–11754. 10.1111/jcmm.15784 32860495 PMC7579702

[B248] Zhang L. Zeyu W. Liu B. Jang S. Zhang Z. Jiang Y. (2021). Pyroptosis in liver disease. Rev. Esp. Enferm. Dig. 113, 280–285. 10.17235/reed.2020.7034/2020 33233902

[B249] Zhang J. Y. Zhou B. Sun R. Y. Ai Y. L. Cheng K. Li F. N. (2021). The metabolite α-KG induces GSDMC-dependent pyroptosis through death receptor 6-activated caspase-8. Cell. Res. 31, 980–997. 10.1038/s41422-021-00506-9 34012073 PMC8410789

[B250] Zhang Y. Yang W. Li W. Zhao Y. (2021a). NLRP3 inflammasome: checkpoint connecting innate and adaptive immunity in autoimmune diseases. Front. Immunol. 12, 732933. 10.3389/fimmu.2021.732933 34707607 PMC8542789

[B251] Zhang Y. Liu W. Zhong Y. Li Q. Wu M. Yang L. (2021b). Metformin corrects glucose metabolism reprogramming and NLRP3 inflammasome-induced pyroptosis *via* inhibiting the TLR4/NF-κB/PFKFB3 signaling in trophoblasts: implication for a potential therapy of Preeclampsia. Oxid. Med. Cell. Longev. 2021, 1806344. 10.1155/2021/1806344 34804360 PMC8601820

[B252] Zhang S. Liang Y. Yao J. Li D. F. Wang L. S. (2022a). Role of pyroptosis in inflammatory bowel disease (IBD): from gasdermins to DAMPs. Front. Pharmacology 13, 833588. 10.3389/fphar.2022.833588 PMC9168461 35677444

[B253] Zhang Q. Tan Y. Zhang J. Shi Y. Qi J. Zou D. (2022). Pyroptosis-related signature predicts prognosis and immunotherapy efficacy in muscle-invasive bladder cancer. Front. Immunol. 13, 782982. 10.3389/fimmu.2022.782982 35479097 PMC9035667

[B254] Zhang S. Pan W. Wang H. Zhi C. Lin Y. Wu P. (2022c). Vitronectin, a novel urinary proteomic biomarker, promotes cell pyroptosis in juvenile systemic lupus erythematosus. Mediat. Inflamm. 2022, 8447675. 10.1155/2022/8447675 PMC9020974 35462789

[B255] Zhang R. N. Jing Z. Q. Zhang L. Sun Z. J. (2023). Epigenetic regulation of pyroptosis in cancer: molecular pathogenesis and targeting strategies. Cancer Lett. 575, 216413. 10.1016/j.canlet.2023.216413 37769798

[B256] Zhang Y. Yu W. Chen M. Zhang B. Zhang L. Li P. (2023). The applications of nanozymes in cancer therapy: based on regulating pyroptosis, ferroptosis and autophagy of tumor cells. Nanoscale 15, 12137–12156. 10.1039/d3nr01722b 37377098

[B257] Zhang K. Luo Z. Zhu X. Yao X. Lu D. Chen L. (2023). Identification of key genes for pyroptosis-induced salivary gland inflammation in sjogren's syndrome based on microarray data and immunohistochemistry analysis. J. Inflamm. Res. 16, 5865–5879. 10.2147/jir.s435008 38076335 PMC10710186

[B258] Zhang W. Liu Z. Zhu J. Liu Z. Zhang Y. Qin G. (2023). Bioorthogonal disruption of pyroptosis checkpoint for high-efficiency pyroptosis cancer therapy. J. Am. Chem. Soc. 145, 16658–16668. 10.1021/jacs.3c04180 37486170

[B259] Zhang L. Bai H. Zhou J. Ye L. Gao L. (2024). Role of tumor cell pyroptosis in anti-tumor immunotherapy. Cell. Insight 3, 100153. 10.1016/j.cellin.2024.100153 38464416 PMC10924176

[B260] Zhang X. Jin L. Wu Y. Huang B. Chen K. Huang W. (2025). Anti-inflammatory properties of biflavonoids derived from Selaginella moellendorffii hieron: targeting NLRP3 inflammasome-dependent pyroptosis. J. Ethnopharmacol. 340, 119172. 10.1016/j.jep.2024.119172 39643022

[B261] Zhang C. Wang Q. Su P. Qian J. Guo Q. Wu W. (2025). NPC1 as a novel therapeutic target for induction of pyroptosis in cancers. Biomark. Res. 13, 115. 10.1186/s40364-025-00823-w 41013744 PMC12465410

[B262] Zhang Y. Ma F. Wang L. Zhu C. Shi J. Da M. (2025). MT1JP/miR-103a-3p induce pyroptosis and regulate the tumor immune microenvironment in gastric cancer. Sci. Rep. 15, 26799. 10.1038/s41598-025-10361-y 40702064 PMC12287438

[B263] Zhang J. Shi M. Sun J. Xu L. Xu Y. Jiang W. (2025). Biodegradable vanadium-based nanomaterials for photothermal-enhanced tumor ferroptosis and pyroptosis. ACS Appl. Mater Interfaces 17, 5735–5751. 10.1021/acsami.4c16568 39818693

[B264] Zhang P. Li Y. Xu P. Zou P. Sheng S. Xiao R. (2025). Identification and exploration of pyroptosis-related genes in macrophage cells reveal necrotizing enterocolitis heterogeneity through single-cell and bulk-sequencing. Int. J. Mol. Sci. 26, 4036. 10.3390/ijms26094036 40362275 PMC12071306

[B265] ZhangL. DaiY. LiQ. JiangX. HeG. (2025). Deciphering the rules of regulated cell death subroutines by *in silico* methods. J. Adv. Res. 10.1016/j.jare.2025.12.016 41419001

[B266] Zhao X. Ma Y. Di J. Qiao Y. Yu J. Yin Y. (2024). Synergetic pyroptosis with apoptosis improving phototherapy of mitochondria-targeted cyanines with superior photostability. ACS Appl. Mater Interfaces 16, 12310–12320. 10.1021/acsami.3c19205 38412031

[B267] Zhao L. Cheng H. Tong Z. Cai J. (2024). Nanoparticle-mediated cell pyroptosis: a new therapeutic strategy for inflammatory diseases and cancer. J. Nanobiotechnology 22, 504. 10.1186/s12951-024-02763-3 39175020 PMC11340130

[B268] Zhao T. Chi Z. Wang D. (2025). Versatility of gasdermin D beyond pyroptosis. Trends Cell. Biol. 35, 1039–1053. 10.1016/j.tcb.2025.02.011 40121145

[B269] Zheng Z. Deng W. Bai Y. Miao R. Mei S. Zhang Z. (2021). The lysosomal rag-ragulator complex licenses RIPK1 and Caspase-8-mediated pyroptosis by Yersinia. Science 372, eabg0269. 10.1126/science.abg0269 35058659 PMC8769499

[B270] Zheng Y. Wang K. Li N. Zhang Q. Chen F. Li M. (2022). Prognostic and immune implications of a novel pyroptosis-related five-gene signature in breast cancer. Front. Surgery 9, 837848. 10.3389/fsurg.2022.837848 PMC9152226 35656090

[B271] Zheng Q. Wang D. Lin R. Chen Y. Xu Z. Xu W. (2023). Quercetin is a potential therapy for rheumatoid arthritis *via* targeting Caspase-8 through ferroptosis and pyroptosis. J. Inflamm. Res. 16, 5729–5754. 10.2147/JIR.S439494 38059150 PMC10697095

[B272] Zhou H. Song W. H. (2023). LncRNA HCG11 accelerates atherosclerosis *via* regulating the miR-224-3p/JAK1 axis. Biochem. Genet. 61, 372–389. 10.1007/s10528-022-10261-0 35931919

[B273] Zhou Z. Wei J. Lu B. Jiang W. Bao Y. Li L. (2021). Comprehensive characterization of pyroptosis patterns with implications in prognosis and immunotherapy in low-grade gliomas. Front. Genet. 12, 763807. 10.3389/fgene.2021.763807 35198000 PMC8859270

[B274] Zhou J. Guo H. Liu L. Feng M. Yang X. Hao S. (2022a). Pyroptosis patterns of colon cancer could aid to estimate prognosis, microenvironment and immunotherapy: evidence from multi-omics analysis. Aging (Albany NY) 14, 7547–7567. 10.18632/aging.204302 36152052 PMC9550258

[B275] Zhou J. Nie R. C. Yin Y. X. Wang Y. Yuan S. Q. Zhao Z. H. (2022b). Genomic analysis uncovers the prognostic and immunogenetic feature of pyroptosis in gastric carcinoma: indication for immunotherapy. Front. Cell. Dev. Biol. 10, 906759. 10.3389/fcell.2022.906759 35912105 PMC9328384

[B276] Zhou S. Liu J. Wan A. Zhang Y. Qi X. (2024). Epigenetic regulation of diverse cell death modalities in cancer: a focus on pyroptosis, ferroptosis, cuproptosis, and disulfidptosis. J. Hematol. Oncol. 17, 22. 10.1186/s13045-024-01545-6 38654314 PMC11040947

[B277] Zhou J. Huang R. Aimaiti M. Zhou Q. Wu X. Zhu J. (2025). Mechanism of RCD and the role of different death signaling pathways in cancer. Biomedicines 13, 1880. 10.3390/biomedicines13081880 40868135 PMC12383337

[B281] Zhou J. Zeng X. Sun J. Yang Y. Wang J. Xiao X. (2026). Gut microbiota-derived indole-3-lactic acid suppresses anti-PD-1 efficacy in esophageal squamous cell carcinoma. Cell Host. Microbe. 34, 639–656. 10.1016/j.chom.2026.02.019 41881017

[B278] Zhu B. Cheng X. Jiang Y. Cheng M. Chen L. Bao J. (2020). Silencing of KCNQ1OT1 decreases oxidative stress and pyroptosis of renal tubular epithelial cells. Diabetes Metab. Syndr. Obes. 13, 365–375. 10.2147/DMSO.S225791 32104033 PMC7025682

[B279] Zhu Y. Xiang Y. Swamiappan S. Li Z. Peng X. (2025). Pyroptosis as a therapeutic target in preeclampsia: current research and future directions. Front. Immunol. 16, 1622550. 10.3389/fimmu.2025.1622550 40636112 PMC12238024

[B280] Zi G. Chen J. Peng Y. Wang Y. Peng B. (2024). Hyperthermia and cisplatin combination therapy promotes caspase-8 accumulation and activation to enhance apoptosis and pyroptosis in cancer cells. Int. J. Hyperth. 41, 2325489. 10.1080/02656736.2024.2325489 38632954

